# CT-Derived Adipose Tissue Biomarkers in Coronary and Carotid Atherosclerosis: A Systematic Review of Dual-Energy, Spectral, and Photon-Counting CT

**DOI:** 10.3390/jcm15207870

**Published:** 2026-10-12

**Authors:** Giuseppe Mauro Bucolo, Michele Gaeta, Alfredo Blandino, Sergio Lucio Vinci, Riccardo Cau, Christian Booz, Thomas Josef Vogl, Vitali Koch, Leon David Gruenewald, Ibrahim Yel, Luca Saba, Silvio Mazziotti, Tommaso D’Angelo

**Affiliations:** 1 Diagnostic and Interventional Radiology Unit, BIOMORF Department, University Hospital “AOU G. Martino”, 98124 Messina, Italy; 2 Departement of Radiology, I.R.C.C.S. Centro Neurolesi Bonino Pulejo, 98124 Messina, Italy; 3 Neuroradiology Unit, Department of Biomedical and Dental Sciences and Morphofunctional Imaging, University Hospital “AOU G. Martino”, 98125 Messina, Italy; 4 Department of Radiology, Azienda Ospedaliero-Universitaria (A.O.U.) di Cagliari, 09124 Cagliari, Italy; 5 Department of Diagnostic and Interventional Radiology, University Hospital of Frankfurt, 60596 Frankfurt, Germany

**Keywords:** epicardial adipose tissue, adipose tissue, atherosclerosis, dual-energy CT, photon counting CT, radiomics, cardiac imaging technique, angiography

## Abstract

**Background/Objectives**: Advanced Computed Tomography (CT) enables multiparametric characterization of local perivascular and regional or systemic adipose tissue, potentially providing markers of vascular inflammation and cardiometabolic remodeling beyond conventional assessment of the vessel lumen and plaque. However, evidence remains fragmented across CT technologies, adipose depots and vascular territories. This systematic review evaluated human studies of adipose-tissue biomarkers derived from dual-layer spectral-detector CT (DLSCT), dual-source dual-energy CT (DS-DECT), rapid kVp-switching dual-energy CT (rsDECT), and photon-counting CT (PCCT) in coronary and carotid atherosclerosis. **Methods**: PubMed and Scopus were systematically searched for English-language human studies published from 1 January 2020, to 13 September 2026, supplemented by backward and forward citation searching of eligible studies and reference-list screening of relevant reviews. Two reviewers independently performed study selection and risk-of-bias assessment. Because of clinical, technical, and methodological heterogeneity, findings were synthesized narratively without meta-analysis. **Results**: Nineteen reports representing 15 likely independent study clusters met the inclusion criteria. DLSCT-derived pericoronary adipose-tissue attenuation; DECT-derived carotid perivascular adipose-tissue parameters; PCCT-derived adipose attenuation, volume, and radiomic features; and spectral measures of hepatic or visceral adiposity were associated with coronary artery disease, stenosis severity, high-risk plaque features, coronary calcification, or symptomatic carotid disease. Reported discrimination estimates were heterogeneous across populations, endpoints, and model structures and were predominantly derived from unvalidated development samples. **Conclusions**: Advanced CT-derived adipose-tissue biomarkers may complement conventional assessment of the vessel lumen and plaque by characterizing local vessel–adipose interactions and systemic cardiometabolic phenotype. However, current measurements are protocol- and platform-dependent, and clinical utility has not been established. Standardized measurement pipelines, biological validation, and prospective multicenter studies are required before routine clinical implementation.

## 1. Introduction

Atherosclerotic cardiovascular disease remains the leading cause of morbidity and mortality worldwide, with coronary artery disease (CAD) and ischemic stroke accounting for a substantial proportion of its clinical burden. Although traditionally characterized by luminal narrowing and plaque morphology, atherosclerosis is now recognized as a chronic lipid-driven inflammatory disease in which persistent vascular inflammation contributes to plaque progression, destabilization, and thrombotic complications [1,2]. Consequently, increasing attention has been directed toward imaging biomarkers capable of characterizing not only the arterial lumen and plaque, but also the biological microenvironment surrounding the vessel wall. Adipose tissue surrounding the heart and major arteries has emerged as an active component of this vascular microenvironment. Epicardial adipose tissue (EAT), located between the myocardium and visceral pericardium, shares the coronary microcirculation and interacts directly with the adjacent myocardium and coronary arteries through paracrine and vasocrine signaling. Pericoronary adipose tissue (PCAT), the EAT immediately surrounding the coronary arteries, and perivascular adipose tissue (PVAT), the broader term describing adipose tissue surrounding blood vessels throughout the body, are increasingly recognized as dynamic regulators of vascular homeostasis. Under physiological conditions these tissues exert metabolic and vasoregulatory functions; however, adipocyte hypertrophy, fibrosis, impaired lipid storage, and a pro-inflammatory secretory phenotype have all been linked to atherosclerotic disease [3,4]. Importantly, this interaction appears bidirectional: dysfunctional adipose tissue may promote vascular inflammation through an outside-in mechanism, whereas inflammatory signals originating from the vessel wall modify adipocyte biology, making PVAT a potential imaging biomarker of local vascular inflammation [5,6]. Coronary CT angiography (CCTA) enables simultaneous evaluation of the coronary lumen, atherosclerotic plaque, and surrounding adipose tissue within a single examination. Conventional CT-derived biomarkers have primarily included adipose tissue volume and mean attenuation, while vessel-specific assessment of PCAT attenuation and the perivascular fat attenuation index (FAI) have provided imaging surrogates of inflammation-related phenotypic changes in perivascular fat. Higher PCAT attenuation has been associated with coronary inflammation, high-risk plaque features, and adverse cardiovascular outcomes, and previous systematic reviews have supported its diagnostic and prognostic value [5,7,8]. Nevertheless, attenuation-based biomarkers remain sensitive to scanner technology, acquisition parameters, reconstruction settings, contrast enhancement, anatomical sampling, and segmentation methodology, limiting comparability across studies and hindering widespread clinical implementation [9,10,11,12]. CT can also characterize broader adipose depots beyond vessel-specific PCAT and carotid PVAT measurements, including whole-heart EAT, periaortic adipose tissue (PAAT), abdominal visceral adipose tissue (VAT), and hepatic fat. These measurements do not represent a single biological construct: EAT retains direct anatomical and functional interactions with the heart and coronary circulation, whereas abdominal VAT and hepatic fat are commonly considered manifestations of visceral or ectopic adiposity associated with broader cardiometabolic risk [3,4,13]. Recent advances in CT technology have substantially expanded the characterization of adipose tissue beyond conventional attenuation measurements. Advanced CT technologies include dual-energy CT (DECT) technologies, such as dual-layer spectral-detector CT (DLSCT), dual-source dual-energy CT (DS-DECT), and rapid kVp-switching dual-energy CT (rsDECT), as well as photon-counting CT (PCCT). Depending on the platform and acquisition or reconstruction approach, these technologies enable quantitative tissue characterization through virtual monoenergetic imaging (VMI), fat fraction, effective atomic number, electron density, iodine-related parameters, and spectral attenuation slopes [14,15,16]. In addition, PCCT offers improved spatial resolution and reduced image noise, facilitating more detailed evaluation of small perivascular adipose depots and supporting quantitative and radiomic analysis of adipose tissue characteristics and heterogeneity [15,17,18]. These technologies have generated a growing number of studies investigating advanced adipose tissue biomarkers in relation to coronary and carotid atherosclerosis [17,19,20,21,22,23]. However, because these biomarkers rely on different physical principles and image reconstruction techniques, they should not be regarded as interchangeable across CT platforms or acquisition protocols [9,14,15,16]. Despite this expanding literature, the available evidence remains fragmented across vascular territories, adipose depots, CT technologies, biomarker definitions, and analytical approaches. Previous reviews have largely focused on conventional CT-derived EAT volume or density, coronary PCAT attenuation, or carotid PVAT within individual vascular territories, without comprehensively integrating advanced spectral and PCCT biomarkers across coronary and carotid atherosclerosis [8,24,25]. To reflect this breadth, we considered adipose biomarkers from anatomically distinct depots, ranging from vessel-adjacent PCAT/PVAT to whole-heart EAT, PAAT, abdominal VAT, and hepatic fat, when directly evaluated in relation to coronary or carotid atherosclerosis; these measurements were treated as complementary rather than interchangeable. Accordingly, this systematic review aimed to synthesize the available human evidence on advanced CT-derived adipose-tissue biomarkers in coronary and carotid atherosclerosis, with emphasis on CT technology, biological rationale, biomarker definition, technical implementation, diagnostic performance, methodological quality, and barriers to clinical translation.

## 2. Materials and Methods

### 2.1. Study Design and Reporting

This systematic review was reported in accordance with the Preferred Reporting Items for Systematic Reviews and Meta-Analyses (PRISMA) 2020 statement [26]. The literature search was reported according to the PRISMA extension for Searching (PRISMA-S) [27]. The review was designed to synthesize human evidence on adipose tissue biomarkers derived from advanced computed tomography techniques in relation to coronary and carotid atherosclerosis. Eligible advanced CT techniques comprised DLSCT, DS-DECT, rsDECT, and PCCT. Because substantial clinical, technical, and methodological heterogeneity was anticipated, results were synthesized narratively without quantitative pooling, following applicable reporting items from the Synthesis Without Meta-analysis (SWiM) guideline [28].

### 2.2. Eligibility Criteria

Eligibility criteria were defined before title and abstract screening. Eligible studies used advanced CT technologies capable of energy-resolved image acquisition or reconstruction, including DLSCT, DS-DECT, rsDECT, and PCCT. Original full-text human studies were eligible if they enrolled adults, derived at least one adipose-tissue biomarker from an advanced CT dataset, and evaluated that biomarker in relation to coronary or carotid atherosclerosis or a directly related imaging or clinical endpoint. Eligible vascular and atherosclerotic endpoints included CAD, coronary plaque presence or composition, high-risk coronary plaque, coronary stenosis severity, coronary artery calcium, carotid plaque vulnerability, symptomatic carotid plaque, and acute ischemic stroke attributable to carotid atherosclerotic disease. Eligible adipose depots and ectopic fat compartments included PCAT, PVAT, EAT, PAAT, VAT, and hepatic fat. Eligible adipose tissue biomarkers comprised attenuation-based measurements (including conventional and VMI attenuation), FAI, fat fraction or fat volume fraction, spectral attenuation slope, effective atomic number, electron density, iodine-related material-decomposition parameters, and radiomic or texture features. Conventional attenuation, volumetric, or radiomic measurements were considered eligible only when derived from an eligible spectral/dual-energy or photon-counting CT acquisition. For cohorts combining eligible advanced CT and conventional CT acquisitions, results had to be separately extractable for the eligible advanced-CT subgroup. Studies evaluating systemic or ectopic adiposity (e.g., hepatic fat or abdominal VAT) were included only when the biomarker was acquired using an eligible advanced CT technology and was directly related to a coronary or carotid atherosclerotic endpoint. Studies were excluded if they were reviews, editorials, letters, commentaries, case reports, conference abstracts lacking a corresponding full-text article, animal studies, phantom studies, ex vivo studies, or technical studies without a clinical or imaging atherosclerotic endpoint. Studies based solely on conventional single-energy CT were excluded, as were studies evaluating plaque composition without an adipose tissue depot or adipose-derived biomarker. Only English-language reports published from 1 January 2020, to 13 September 2026, were considered eligible.

### 2.3. Information Sources and Search Strategy

PubMed (National Library of Medicine) and Scopus (Elsevier) were systematically searched on 8 June 2026, for reports published from 1 January 2020, to the search date. The searches were subsequently updated through 13 September 2026, retaining the original structure. The January 2020 starting date was selected pragmatically to focus the review on contemporary implementations of dual-energy, spectral, and photon-counting CT and their associated reconstruction and post-processing approaches. Embase was not included in the original search workflow, and Web of Science was unavailable through institutional access. Three complementary strategies were applied in each database. The first combined core terms for adipose tissue, atherosclerosis, and advanced CT; the second expanded the adipose and plaque-vulnerability terminology; and the third was a high-sensitivity radiomics and plaque-characterization strategy to identify reports in which adipose tissue assessment might not appear in the title or abstract. Search concepts included PCAT, PVAT, EAT, VAT, FAI, adipose tissue, atherosclerosis, CAD, coronary and carotid plaque, plaque vulnerability, DECT, DLSCT, DS-DECT, rsDECT, VMI, effective atomic number, and PCCT. PubMed searches used title and abstract fields, whereas Scopus searches used TITLE-ABS-KEY. Complete database-specific strategies and the initial-search log are provided in Supplementary Table S1; operational details of the database update and citation searching are reported in Supplementary Section S1.7. After the database update, 18 reports were eligible and served as the seed set for citation searching. Backward citation searching was performed by screening the reference lists of these 18 reports and of the relevant reviews by Sagris et al. [8], Mancio et al. [24], and Tuttolomondo et al. [25]. Forward citation searching was performed in Scopus for the same 18 reports. Records identified through citation searching were deduplicated across sources and checked against previously identified records before screening according to the unchanged eligibility criteria.

### 2.4. Selection Process

Records retrieved from PubMed and Scopus were exported in NBIB and RIS formats, respectively, and imported into Zotero (version 9.0.6; Corporation for Digital Scholarship, Falls Church, VA, USA) for duplicate removal. After deduplication, unique records were imported into Rayyan (Rayyan Systems Inc., Cambridge, MA, USA) for screening [29]. Two reviewers independently screened titles and abstracts against the eligibility criteria and independently assessed potentially eligible full-text reports. Before finalizing study selection, two records initially excluded during title and abstract screening were independently reassessed by both reviewers and advanced to full-text assessment by consensus; the eligibility criteria were not modified. All disagreements at either screening stage were resolved by consensus after re-examination of the record or report. Full-text eligibility was confirmed by verifying the study population, CT technology, adipose tissue depot and biomarker, vascular territory, endpoint, and study design. Full-text exclusions and the corresponding reasons are reported in Supplementary Table S2.

### 2.5. Data Collection Process and Data Items

Data were collected in a structured Microsoft Excel workbook (Microsoft Excel for Microsoft 365, version 2609; Microsoft Corporation, Redmond, WA, USA). One reviewer extracted the data, and a second reviewer verified every entry against the full-text report and any available supplementary material. Discrepancies were resolved by consensus after re-examination of the original source. Study-level data included first author, publication year, journal, country and center, study design, participant characteristics, sample size, and unit of analysis. Technical data included scanner and CT technology, acquisition and reconstruction approach, contrast phase when applicable, adipose depot, anatomical measurement site, segmentation method, attenuation threshold or fat definition, extracted biomarkers, post-processing software, reader procedures, and reproducibility estimates. Outcome data included the atherosclerotic endpoint, direction and magnitude of association, group comparisons, regression estimates, odds ratios (OR), confidence intervals (CI), *p* values, diagnostic or predictive performance, thresholds, calibration, internal validation, and external validation when reported. For radiomics studies, the extracted items additionally included segmentation strategy, feature-extraction software, feature-reduction or selection approach, model-development procedure, and validation method. All review-relevant outcomes, associations, and performance estimates reported for eligible adipose biomarkers were sought. When a report presented multiple related results, the result corresponding to the authors’ stated primary objective or endpoint and most directly addressing the review question was selected; results were not selected solely on the basis of statistical significance. During data synthesis, single adipose biomarkers and final multivariable adipose-containing models were considered separately for the descriptive AUC display, with no more than one representative estimate of each type selected per report. When multiple eligible estimates were available, the estimate most closely aligned with the report’s primary objective and the review question was selected; for multivariable analyses, the authors’ final adipose-containing model was used, with external-validation estimates preferred when available. When both development and external-validation estimates were available, the external-validation estimate was selected. Reported 95% confidence intervals were displayed when available and were not reconstructed when omitted from the original report. When numerical values were reported only in tables or supplementary material, they were extracted from those sources. No unreported confidence intervals, missing performance measures, or validation statistics were estimated or imputed. During data extraction, potential overlap between reports was assessed by comparing authorship, institution, recruitment setting, study period, CT platform, sample size, eligibility criteria, endpoint definition, and statistical modeling strategy. When reports were judged to belong to a likely overlapping study cluster, all reports were retained for transparency but were not treated as independent replications when summarizing the evidence base. Corresponding authors were not contacted for participant-level clarification; overlap was therefore considered likely rather than confirmed.

### 2.6. Risk of Bias Assessment

Risk of bias and concerns regarding applicability were assessed with official study-design-specific tools. Following full-text characterization, each report was assigned to the relevant appraisal tool according to its primary review-relevant analytical aim; where multiple analysis types were reported, this aim, rather than the study-design label used by the authors, guided classification. Reports whose primary objective was the development of a multivariable diagnostic or prognostic prediction model were assessed using the original Prediction model Risk Of Bias ASsessment Tool (PROBAST) (version dated 15 May 2019), including the Participants, Predictors, Outcome, and Analysis domains [30,31]. A specific diagnostic-accuracy estimate was assessed using Quality Assessment of Diagnostic Accuracy Studies-3 (QUADAS-3), version 1.2, with judgments made for the selected accuracy estimate and its applicability to the review question [32]. Case–control studies were appraised using the 2020 JBI Critical Appraisal Checklist for Case Control Studies [33]. Analytical cross-sectional studies were assessed using the revised JBI critical appraisal tool for risk of bias in analytical cross-sectional studies [34]. For Chen et al. [35] and Chen et al. [19], QUADAS-3 was applied to the CT40keV and PVAT40keV estimates, respectively, as these were the diagnostic-accuracy estimates selected for the review synthesis. Other analyses within these reports were not treated as separate diagnostic-accuracy synthesis units and were not assessed separately with QUADAS-3. Two reviewers independently completed all assessments and resolved disagreements by consensus after consulting the full text, supplementary material, and official guidance. PROBAST and QUADAS-3 findings were reported using their official domain-level and overall judgments. JBI assessments were reported item by item as Yes, No, Unclear, or Not Applicable, and no non-validated numerical score or overall low-, moderate-, or high-risk category was assigned. For radiomics reports, segmentation, feature extraction, feature selection, and validation were recorded as methodological descriptors and considered only within the applicable domains or items of the selected official tool. No additional signaling questions, hybrid risk-of-bias framework, or radiomics quality score was introduced. Complete domain- and item-level assessments are reported in Supplementary Tables S3–S6.

### 2.7. Synthesis Methods

After data extraction, the reports were organized into four clinically and technically coherent technology–biomarker groups: (1) coronary DLSCT-derived PCAT attenuation or FAI; (2) carotid DECT-derived PVAT parameters; (3) PCCT-based adipose-tissue attenuation, volume, and radiomics/texture features; and (4) systemic or ectopic adiposity assessed using DECT. Reports were synthesized within these groups without assigning a formal evidence hierarchy or quantitative weight to any adipose depot. For descriptive purposes, eligible adipose biomarkers were classified according to their mode of derivation as conventional quantitative measurements derived from eligible advanced CT datasets, spectral or material-specific quantitative measurements, radiomic/texture features, or adipose-containing multivariable models. Clinical and methodological comparability was evaluated after extraction by comparing the population, vascular territory, CT platform, adipose depot, biomarker definition, endpoint, reference standard, unit of analysis, and reported statistical estimand. A quantitative meta-analysis was not performed because no sufficiently homogeneous group of studies evaluated a common biomarker, endpoint, reference standard, unit of analysis, and statistical estimand. Accordingly, AUCs, sensitivities, specificities, odds ratios, regression estimates, and cut-off values were extracted and presented descriptively but were not pooled. Reported sample sizes were summarized using the range, and categorical study characteristics were summarized using counts. Because probable participant overlap could not be quantified across the Chen-Dang-Zhu coronary cluster and the three Zhang carotid reports, no aggregate participant total or cluster-level median sample size was calculated. The study-level characteristics supporting these overlap judgments are provided in Supplementary Table S7. The narrative synthesis emphasized the direction and magnitude of associations; the relative performance of spectral, attenuation, volumetric, and radiomic biomarkers; the incremental contribution of adipose biomarkers within combined models; and the presence or absence of calibration and validation. Reported confidence intervals were presented when available and were not reconstructed when omitted from the original report. Study characteristics, technical methods, and principal findings were summarized descriptively and in structured tables. An evidence map was used to summarize the distribution of reports across the four technology-biomarker groups. Risk-of-bias and critical-appraisal judgments were summarized in a traffic-light plot. All eligible AUC estimates and their selection status are reported in Supplementary Table S8. Selected study-level AUCs were displayed descriptively in a two-panel plot, separating single adipose biomarkers from multivariable adipose-containing models. Reported 95% confidence intervals were shown when available, and no pooled summary estimate was provided. No inferential comparison between studies, formal heterogeneity statistic, or statistical weighting according to risk of bias was performed. Risk of bias due to missing results was not formally assessed because no common outcome-specific synthesis or meta-analysis was undertaken and the number of comparable reports per endpoint was insufficient. The certainty of evidence was not formally graded because the review combined heterogeneous association studies, diagnostic-accuracy estimates, and prediction-model studies without a common estimand.

### 2.8. Protocol and Registration

The review was not prospectively registered in PROSPERO or another registry, and no formal review protocol was prepared or published.

## 3. Results

**Figure 1:**
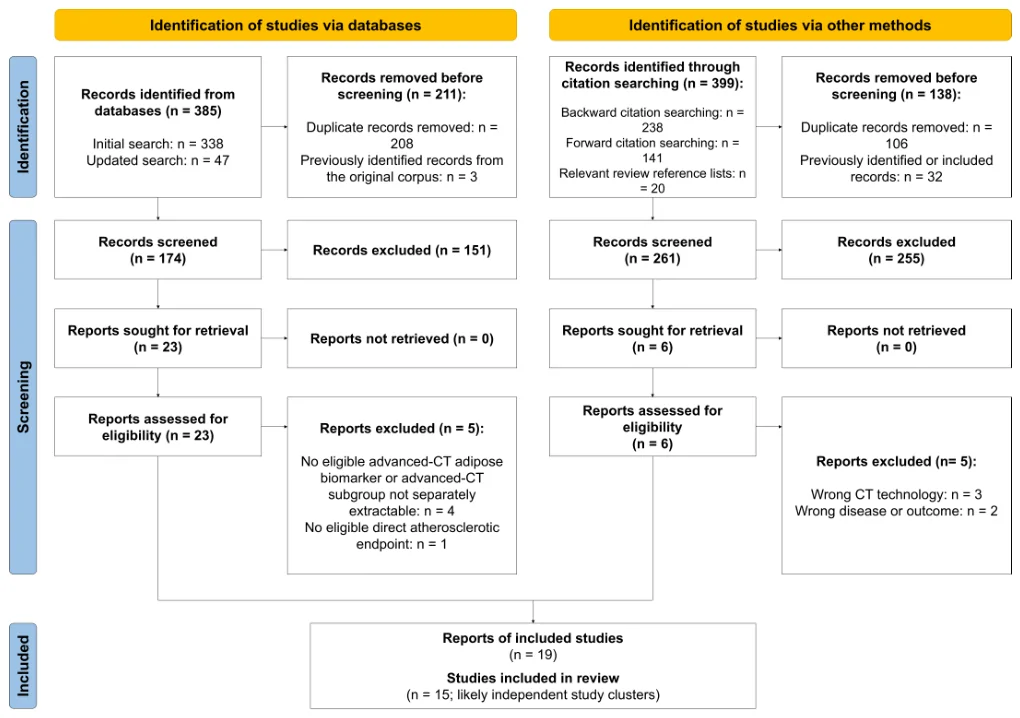
Preferred Reporting Items for Systematic Reviews and Meta-Analyses, or PRISMA, flow diagram.

The study-selection process is summarized in Figure 1. The initial database search identified 338 records (114 from PubMed and 224 from Scopus). After removal of 182 duplicate records, 156 unique records underwent title and abstract screening, of which 134 were excluded. Twenty-two reports were sought for retrieval; all were retrieved and assessed for eligibility. Five reports were excluded: four did not provide an eligible advanced-CT adipose biomarker or a separately extractable advanced-CT subgroup, and one evaluated PCCT-derived carotid PVAT without relating it to an eligible atherosclerotic imaging or clinical endpoint. The excluded full-text reports are listed with individual reasons in Supplementary Table S2. Seventeen reports were included from the initial database search. The updated database search, conducted through 13 September 2026, identified 47 records. After removal of 26 duplicates, 21 records remained; three had already been identified in the original corpus, leaving 18 new records for title and abstract screening. Seventeen were excluded, and one report underwent full-text assessment and was included [23]. Thus, across the initial and updated database searches, 385 records were identified, 174 unique records underwent title and abstract screening, 23 reports were assessed for eligibility, and 18 reports were included. Citation searching identified 399 candidate records, comprising 238 records from backward citation searching, 141 from forward citation searching in Scopus, and 20 from reference-list screening of relevant reviews. After removal of 106 duplicate records and 32 records already identified or included, 261 new records underwent title and abstract screening; 255 were excluded. Six reports were sought for retrieval and assessed for eligibility; five were excluded and one additional report was included [18]. Overall, 19 eligible reports were included in the qualitative synthesis, representing 15 likely independent study clusters. Chen et al. [35], Dang et al. [21], and Zhu et al. [36] were retained as separate reports because they addressed high-risk plaque, CAD presence and severity, and significant stenosis, respectively; however, their shared center, scanner platform, ethics approval, and overlapping recruitment periods indicated a likely overlapping coronary DLSCT cohort [21,35,36]. The three Zhang et al. [20,22,37] reports were likewise retained separately because they evaluated different combinations of carotid intraplaque, lumen, PVAT, and serum lipid parameters, but were counted as one likely overlapping carotid DS-DECT cluster.

### 3.1. Characteristics of the Included Reports

**Figure 2:**
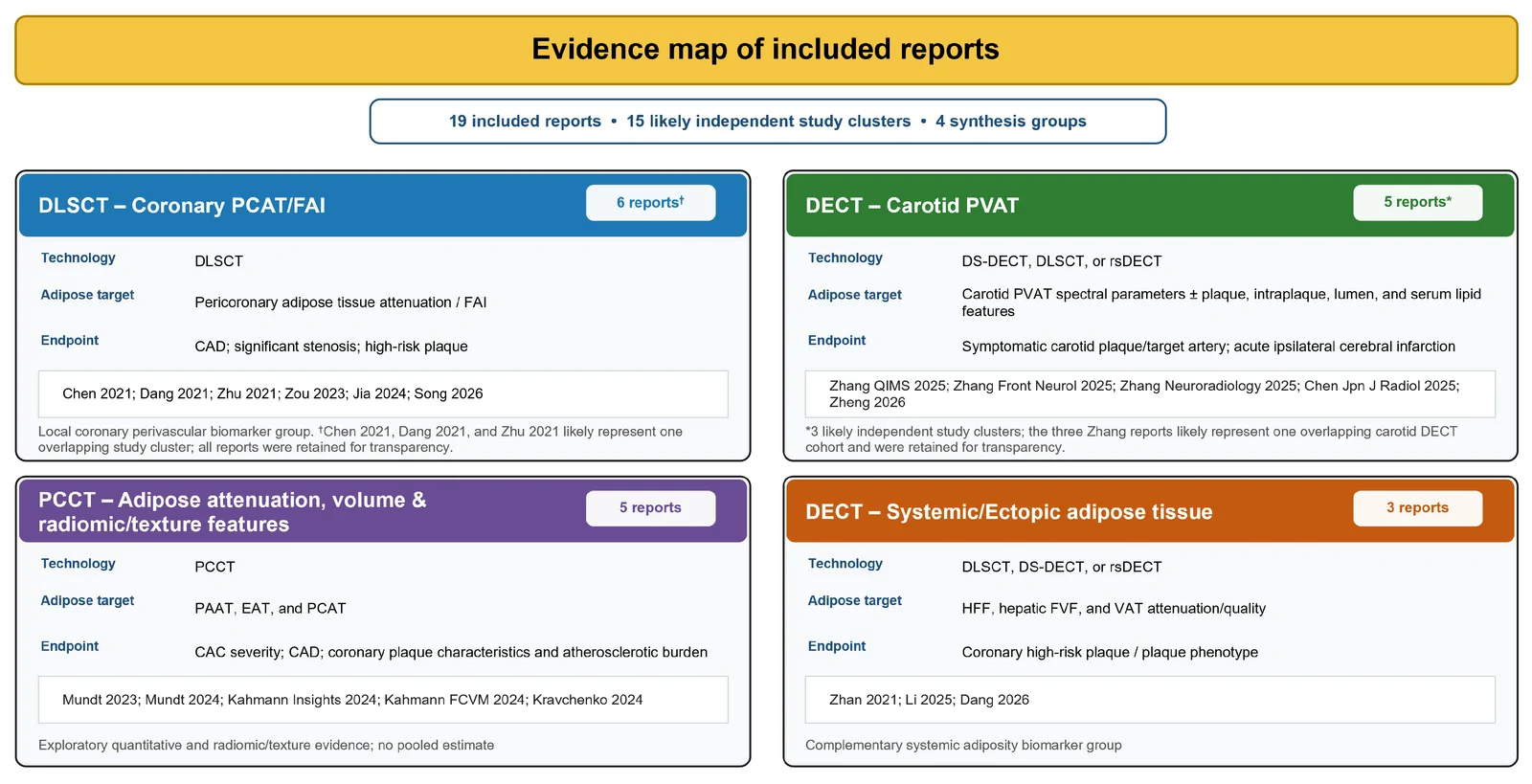
Evidence map of the 19 included reports organized into four technology–biomarker synthesis groups [17,18,19,20,21,22,23,35,36,37,38,39,40,41,42,43,44,45,46]. Reports were grouped to support structured narrative synthesis without quantitative pooling. Abbreviations: CAC, coronary artery calcium; CAD, coronary artery disease; DECT, dual-energy computed tomography; DLSCT, dual-layer spectral-detector computed tomography; DS-DECT, dual-source dual-energy computed tomography; EAT, epicardial adipose tissue; FAI, fat attenuation index; FVF, fat volume fraction; HFF, hepatic fat fraction; PAAT, periaortic adipose tissue; PCAT, pericoronary adipose tissue; PCCT, photon-counting computed tomography; PVAT, perivascular adipose tissue; rsDECT, rapid kVp-switching dual-energy computed tomography; VAT, visceral adipose tissue.

The characteristics of the included reports are summarized in Table 1. The 19 reports were published between 2021 and 2026; 14 originated from China, 4 from Germany, and 1 from the USA. Reported sample sizes ranged from 36 to 403 participants. Because the included reports used patient-, vessel-, and plaque-level analyses and two sets of publications likely shared participants, no aggregate participant count or cluster-level median sample size was calculated. Six reports evaluated coronary DLSCT-derived PCAT attenuation or FAI, five reports evaluated carotid DECT-derived PVAT parameters, five reports examined PCCT-based adipose-tissue attenuation, volume, and radiomic/texture features, and three reports investigated systemic or ectopic adiposity using DLSCT, DS-DECT, or rsDECT. An evidence map of the reports across these technology–biomarker groups is shown in Figure 2.

For methodological appraisal, six reports were classified as prediction-model studies, two as diagnostic-accuracy studies, four as case–control studies, and seven as analytical cross-sectional studies. Eighteen reports were single-center investigations, whereas one was a dual-center study that included an external-validation cohort.

### 3.2. CT Technologies and Adipose Tissue Assessment

Technical acquisition, reconstruction, biomarker extraction, software, reproducibility characteristics, and biomarker derivation categories are summarized in Table 2.

All six coronary PCAT reports used the Philips IQon DLSCT platform and evaluated conventional or 120-kVp-equivalent reconstructions together with low-keV VMI, although the anatomical sampling strategy varied from proximal-vessel to plaque-centered or peri-plaque measurements [21,35,36,38,39,40].

Three carotid PVAT reports used DS-DECT; the dual-center report used SOMATOM Definition Flash for training and SOMATOM Drive for external validation [20]. The fourth report used Philips IQon DLSCT [19]. Across the DS-DECT reports, PVAT spectral and material-decomposition parameters were integrated with plaque, lumen, or serum lipid variables, whereas the DLSCT report assessed plaque-centered PVAT attenuation on 40- and 70-keV VMI and polyenergetic images [19,20,22,37]. A fifth carotid report by Zheng et al. used rapid kVp-switching dual-energy CTA on a GE Revolution Apex scanner, combining PVAT attenuation and material-decomposition parameters with plaque and stenosis measurements [23].

All five PCCT reports were performed using the first-generation Siemens NAEOTOM Alpha platform, but the adipose tissue targets and analytic approaches differed across studies and included thoracic PAAT radiomics, whole EAT radiomics and quantitative measures, coronary plaque combined with conventional EAT measures, left anterior descending artery (LAD) and right coronary artery (RCA) PCAT radiomics, and quantitative EAT volume and attenuation measurements [17,18,41,42,43].

**Table 1:** Characteristics of the included reports.

Study	Design/Population	Sample/Unit of Analysis	CT Technique	Adipose Biomarker	Primary Endpoint
**Chen et al., 2021 [35] (QIMS) ** ^a^	China; retrospective single-center study of consecutive patients with chest pain undergoing CCTA	104 patients; 220 plaques; patient- and plaque-level analyses	DLSCT CCTA (Philips IQon)	Plaque-centered PCAT attenuation on conventional and monoenergetic images, spectral attenuation slope, and effective atomic number; EAT volume and attenuation were secondary measures	CCTA-defined high-risk coronary plaque
**Dang et al., 2021 [21] (Front Cardiovasc Med)** ^a^	China; retrospective single-center analytical cross-sectional study of patients with chest pain and suspected CAD undergoing CCTA	403 patients; patient-level analysis by CAD presence and stenosis category	DLSCT CCTA (Philips IQon)	Lesion-centered FAI120kVp, FAI40keV, and FAI70keV, spectral attenuation slope, and effective atomic number; EAT attenuation was secondary	Presence of CAD and coronary stenosis severity
**Zhu et al., 2021 [36] (J Cardiothorac Surg)** ^a^	China; retrospective single-center selected case–control study; patients sampled 1:1:1 according to coronary stenosis category	99 patients (33 no stenosis; 33 <50% stenosis; 33 ≥50% stenosis); patient-level analysis	DLSCT CCTA (Philips IQon)	Lesion-centered FAI40keV, spectral attenuation slope, and effective atomic number; EAT volume was secondary	Coronary stenosis category and significant stenosis (≥50%)
**Zou et al., 2023 [38] (QIMS)**	China; retrospective cross-sectional study with 1:1 propensity-score matching of patients with suspected CAD	90 matched patients (45 CAD; 45 non-CAD); 270 vessels; patient- and vessel-level analyses	DLSCT CCTA (Philips IQon)	Proximal three-vessel FAI120kVp, FAIVMI and spectral attenuation slopes	Presence of CAD and vessel-level coronary plaque
**Jia et al., 2024 [39] (Front Med)**	China; retrospective single-center analytical cross-sectional study; suspected coronary atherosclerosis	160 patients; 231 stenotic plaque-bearing coronary arteries; vessel-level analysis	DLSCT CCTA (Philips IQon)	Proximal and peri-plaque FAICI and FAIVMI, spectral attenuation slopes, and additional peri-plaque spectral parameters	Coronary stenosis severity and plaque type
**Song et al., 2026 [40] (BMC Med Imaging)**	China; retrospective single-center study of consecutive patients with known or suspected coronary atherosclerotic heart disease	182 patients; 312 plaques overall; HRP analysis included 127 patients and 186 plaques; plaque-level analysis	DLSCT CCTA (Philips IQon)	Peri-plaque FAI120kVp and FAI40–100keV, spectral attenuation slopes, and effective atomic number	Plaque calcification category and CCTA-defined high-risk plaque
**Zhang et al., 2025 [22] (QIMS)** ^b^	China; retrospective single-center prediction-model development study of patients undergoing carotid CTA and cranial MRI	69 patients (STA 20; ATA 49); patient-level analysis	Third-generation DS-DECT carotid CTA (Siemens SOMATOM Drive)	Carotid PVAT spectral parameters integrated with plaque and lumen variables	Ipsilateral anterior-circulation acute cerebral infarction on MRI-DWI/ADC
**Zhang et al., 2025 [37] (Front Neurol)** ^b^	China; retrospective single-center prediction-model development study of consecutive patients undergoing carotid CTA and cranial MRI	75 patients (STA 24; ATA 51); patient-level analysis	Third-generation DS-DECT carotid CTA (Siemens SOMATOM Drive)	Carotid PVAT and intraplaque spectral parameters	Ipsilateral anterior-circulation acute cerebral infarction on MRI-DWI/ADC
**Zhang et al., 2025 [20] (Neuroradiology)** ^b^	China; retrospective dual-center prediction-model development and external-validation study of patients with suspected acute cerebrovascular events undergoing carotid CTA and cranial MRI	212 patients: training cohort 137 (STA 61; ATA 76) and external-validation cohort 75 (STA 26; ATA 49); patient-level analysis	Second- and third-generation DS-DECT carotid CTA (Siemens SOMATOM Definition Flash, training; SOMATOM Drive, external validation)	Carotid PVAT fat fraction, iodine concentration, and 40-keV attenuation integrated with plaque-volume measures, intraplaque spectral parameters, and serum lipid biomarkers	Ipsilateral anterior-circulation acute cerebral infarction on MRI-DWI/ADC
**Chen et al., 2025 [19] (Jpn J Radiol)**	China; prospective single-center observational study of patients with extracranial carotid atherosclerotic stenosis	73 patients; 73 selected plaques (29 symptomatic; 44 asymptomatic); one plaque per patient	DLSCT carotid CTA (Philips IQon)	Plaque-centered carotid PVAT attenuation on 40- and 70-keV monoenergetic and polyenergetic images	Identification of symptomatic carotid plaque
**Zheng et al., 2026 [23] (Insights Imaging)**	China; retrospective single-center study of consecutive adults with CTA-confirmed carotid atherosclerosis; prediction-model development	306 patients (169 symptomatic; 137 asymptomatic); patient-level analysis	rsDECT carotid CTA (GE Revolution Apex)	Carotid PVAT attenuation on conventional and 40-/70-keV images, fat fraction, iodine concentration, effective atomic number, and spectral attenuation slope, integrated with plaque and stenosis measures	Symptomatic carotid plaque status (ipsilateral ischemic stroke, TIA, or amaurosis fugax within 6 months before CTA)
**Mundt et al., 2023 [17] (BMC Med Imaging)**	Germany; retrospective single-center analytical cross-sectional radiomics study	55 patients; patient-level analysis by Agatston-score group	First-generation PCCT (Siemens NAEOTOM Alpha)	Thoracic periaortic adipose tissue radiomics	Coronary calcium severity (Agatston score)
**Mundt et al., 2024 [41] (Diagnostics)**	Germany; retrospective single-center analytical cross-sectional radiomics study	53 patients; patient-level analysis by Agatston-score group	First-generation PCCT (Siemens NAEOTOM Alpha)	EAT radiomics, density, and volume	Coronary calcium severity (Agatston score)
**Kahmann et al., 2024 [42] (Insights Imaging)**	Germany; retrospective single-center analytical cross-sectional study of plaque-positive patients without ischemic heart disease	61 patients; 306 plaques; patient- and plaque-level analyses	First-generation PCCT (Siemens NAEOTOM Alpha)	EAT density and thickness integrated with coronary plaque radiomics	High-risk plaque, ≥50% and ≥70% stenosis, triple-vessel disease, and combined risk phenotype
**Kahmann et al., 2024 [43] (Front Cardiovasc Med)**	Germany; retrospective matched case–control radiomics study of patients with CAD confined to the left coronary artery and matched non-CAD controls	36 patients (18 CAD; 18 non-CAD); LAD- and RCA-level PCAT analyses	First-generation PCCT (Siemens NAEOTOM Alpha)	LAD and RCA PCAT radiomics	Presence of CAD/coronary plaque
**Kravchenko et al., 2024 [18] (Eur J Radiol)**	USA; prospective single-center paired comparison study of consecutive patients undergoing clinically indicated CCTA for stable chest pain or TAVR planning	50 patients; patient-level paired analysis; PCCT within 30 days of EID-CT	PCCT CCTA (Siemens NAEOTOM Alpha); EID-CT comparator (Siemens SOMATOM Force)	Whole-EAT volume and mean attenuation	Intra-individual comparison of EAT volume and attenuation between PCCT and EID-CT
**Zhan et al., 2021 [44] (Jpn J Radiol)**	China; matched case–control study of patients with NAFLD undergoing dual-energy CCTA	172 patients (59 no plaque; 59 non-HRP; 54 HRP); patient-level analysis	Third-generation DS-DECT CCTA (Siemens SOMATOM Force)	Hepatic fat fraction; EAT and total PCAT volume and attenuation were secondary measures	CCTA-defined high-risk coronary plaque
**Li et al., 2025 [46] (Clin Radiol)**	China; retrospective matched case–control study of patients with NAFLD undergoing CCTA and abdominal contrast-enhanced spectral CT	159 patients (57 no plaque; 54 non-HRP; 48 HRP); patient-level analysis	Conventional CCTA and rsDECT abdominal contrast-enhanced CT (GE Revolution CT)	Hepatic fat volume fraction; EAT and total PCAT volume and attenuation were secondary measures	CCTA-defined high-risk coronary plaque
**Dang et al., 2026 [45] (Insights Imaging)**	China; single-center cross-sectional observational study with prospective consecutive enrollment of patients with suspected CAD; MetS comparison	392 patients (182 MetS; 210 non-MetS); HRP analysis restricted to the MetS group; patient-level analysis	DLSCT CCTA and non-contrast abdominal CT (Philips IQon)	L3 VAT area, 40- and 70-keV attenuation, spectral attenuation slope, and effective atomic number	CCTA-defined high-risk coronary plaque in patients with MetS

*Note*. ^a^ Chen et al. (2021) [35], Dang et al. (2021) [21], and Zhu et al. (2021) [36] were considered one likely overlapping coronary DLSCT study cluster. ^b^ The three Zhang et al. (2025) [20,22,37] reports (QIMS, Frontiers in Neurology, and Neuroradiology) were considered one likely overlapping carotid DS-DECT study cluster. All reports were retained for transparency, but reports within these clusters were not treated as independent replications. Abbreviations: ADC, apparent diffusion coefficient; ATA, asymptomatic target artery; CAD, coronary artery disease; CCTA, coronary computed tomography angiography; CTA, computed tomography angiography; DLSCT, dual-layer spectral-detector computed tomography; DS-DECT, dual-source dual-energy computed tomography; DWI, diffusion-weighted imaging; EID-CT, energy-integrating detector computed tomography; EAT, epicardial adipose tissue; FAI, fat attenuation index; HRP, high-risk plaque; keV, kiloelectron volt; LAD, left anterior descending artery; MetS, metabolic syndrome; MRI, magnetic resonance imaging; NAFLD, non-alcoholic fatty liver disease; PCAT, pericoronary adipose tissue; PCCT, photon-counting computed tomography; PVAT, perivascular adipose tissue; RCA, right coronary artery; rsDECT, rapid kVp-switching dual-energy computed tomography; STA, symptomatic target artery; TAVR, transcatheter aortic valve replacement; TIA, transient ischemic attack; VAT, visceral adipose tissue.

**Figure 3:**
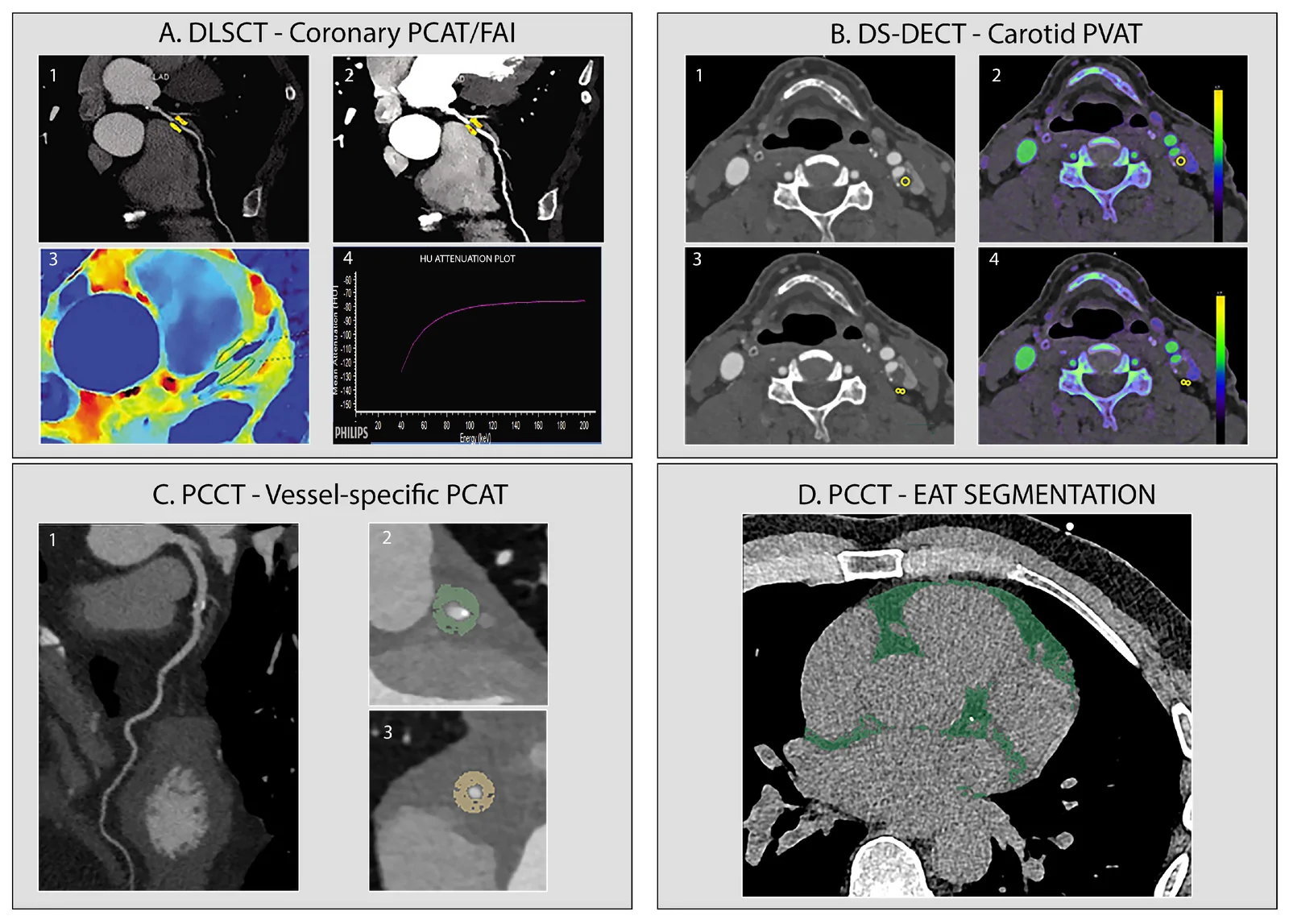
Representative advanced CT approaches for adipose-tissue assessment in the included studies. (**A1**–**A4**) Coronary plaque-centred pericoronary adipose tissue assessment on dual-layer spectral-detector CT (DLSCT), using conventional 120-kVp imaging (**A1**), 40-keV virtual monoenergetic imaging (**A2**), effective atomic number mapping (**A3**), and the corresponding spectral attenuation curve (**A4**). Adapted from Dang et al. [21]. (**B1**–**B4**) Carotid plaque and plaque-adjacent perivascular adipose tissue assessment on dual-source dual-energy CT (DS-DECT). Plaque regions of interest are shown on virtual non-contrast and Rho/Z spectral reconstructions (**B1**,**B2**), while the corresponding plaque-adjacent perivascular adipose tissue regions of interest are shown in (**B3**,**B4**). Adapted from Zhang et al. [37]. (**C1**–**C3**) Vessel-specific pericoronary adipose tissue assessment on photon-counting CT (PCCT), including curved multiplanar reconstruction of the left anterior descending artery (**C1**) and segmentation of left anterior descending and right coronary artery pericoronary adipose tissue (**C2**,**C3**) for radiomic feature extraction. Adapted from Kahmann et al. [43]. (**D**) Whole epicardial adipose tissue segmentation on PCCT, with the segmented adipose compartment shown in green. Adapted from Mundt et al. [41]. Panels (**A**–**D**) were adapted from articles published under the Creative Commons Attribution 4.0 International License (CC BY 4.0). Images were cropped, resized, relabelled, and assembled into a new multipanel figure.

Representative implementations of coronary PCAT, carotid plaque-adjacent PVAT, vessel-specific PCAT, and whole-EAT assessment in the included studies are shown in Figure 3 [21,37,41,43].

Formal numerical repeatability or agreement was reported in eight reports: for PCAT by Chen et al. [35], Dang et al. [21], and Zhu et al. [36]; for carotid PVAT attenuation by Chen et al. [19]; for plaque and PVAT spectral measurements by Zheng et al. [23]; for peri-plaque spectral measurements by Song et al. [40]; for hepatic and cardiac adipose measurements by Zhan et al. [44]; and for L3-level VAT parameters by Dang et al. [45]. Kravchenko et al. [18] additionally assessed paired agreement between CT systems and reconstructions using correlation and Bland–Altman analyses, which was distinguished from reader repeatability. Other reports used consensus reading or expert review, or did not report a reliability coefficient.

**Table 2:** Technical characteristics of CT acquisition and adipose tissue assessment.

Study	Scanner/Acquisition	Reconstruction	Biomarker Extraction	Biomarker Derivation Category	Image Analysis/Reproducibility
**Chen et al., 2021 [35] (QIMS)** ^a^	Philips IQon; prospectively ECG-triggered DLSCT CCTA	Polychromatic images, 40- and 70-keV VMI, and Zeff maps	EAT threshold −190 to −30 HU; plaque-centered PCAT within one vessel diameter with energy-specific thresholds; CTpoly, CT40keV, CT70keV, spectral slope, and Zeff	Conventional quantitative Spectral/material-specific quantitative	Cardiac Risk Assessment v1.2.0 and syngo.via VB20; two readers; PCAT interobserver ICC 0.816
**Dang et al., 2021 [21] (Front Cardiovasc Med)** ^a^	Philips IQon; prospectively ECG-gated DLSCT CCTA (Step & Shoot; 120 kVp)	Spectral level 4; 0.9-mm-thick/0.45-mm-increment images; polychromatic 120-kVp images, 40- and 70-keV VMI, and Eff-Z maps	Plaque-centered PCAT around the maximal stenosis with energy-specific thresholds; FAI120kVp, FAI40keV, FAI70keV, spectral slope, and Eff-Z; EAT attenuation (−190 to −30 HU) was secondary	Conventional quantitative Spectral/material-specific quantitative	Dr. Wise V200831, IntelliSpace Portal v6.5, and Cardiac Risk Assessment v1.2.0; blinded single reader, triplicate measurements; 60-case intraobserver ICCs >0.75
**Zhu et al., 2021 [36] (J Cardiothorac Surg)** ^a^	Philips IQon; prospectively ECG-gated DLSCT CCTA (Step & Shoot; 120 kVp)	Spectral level 4; 0.9-mm-thick/0.45-mm-increment images; 40- and 70-keV VMI, conventional images, and Eff-Z maps	Lesion-centered PCAT in stenosis groups versus proximal 40-mm RCA PCAT in controls, within one vessel diameter; energy-specific thresholds; FAI40keV, spectral slope, and Eff-Z; EAT volume was secondary	Conventional quantitative Spectral/material-specific quantitative	IntelliSpace Portal v6.5 and Heart Disease Risk Assessment v1.2.0; two readers with averaged values; three repeated measurements; reported intraobserver kappa 0.848
**Zou et al., 2023 [38] (QIMS)**	Philips IQon; retrospectively ECG-gated DLSCT CCTA	120-kVp-equivalent images and 40–100-keV VMI	Semiautomatic proximal 40-mm RCA/LAD/LCX PCAT with energy-specific fat thresholds; FAI and spectral slopes	Conventional quantitative Spectral/material-specific quantitative Adipose-containing multivariable model	IntelliSpace Portal Vision 10 and ShuKun software (ShuKun Technology, Beijing, China); no formal reliability coefficient
**Jia et al., 2024 [39] (Front Med)**	Philips IQon; retrospectively ECG-gated DLSCT CCTA	Conventional images, 40–100-keV VMI, VNC, and Zeff maps	Semiautomatic proximal and peri-plaque PCAT; manual peri-plaque VNC/Zeff ROIs; FAI and spectral slopes	Conventional quantitative Spectral/material-specific quantitative	IntelliSpace Portal Vision 10 and ShuKun software (ShuKun Technology, Beijing, China); no formal repeatability assessment
**Song et al., 2026 [40] (BMC Med Imaging)**	Philips IQon; DLSCT CCTA	120-kVp-equivalent images, 40–100-keV VMI, and Eff-Z maps	Plaque-centered cylindrical PCAT VOI with energy-specific thresholds; FAI, spectral slopes, and Eff-Z	Conventional quantitative Spectral/material-specific quantitative Adipose-containing multivariable model	CoronaryDoc and IntelliSpace Portal 10.1.5; inter- and intraobserver ICCs 0.824–0.939
**Zhang et al., 2025 [22] (QIMS)** ^b^	Siemens SOMATOM Drive; third-generation DS-DECT head-and-neck CTA at 80/140 kVp	0.6-mm Br40 reconstructions; VNC, Rho/Z, and 40- and 90-keV monoenergetic images	Two PVAT ROIs adjacent to the most prominent carotid plaque and at least 1 mm from vessel edges/soft tissue; FF, VNC, IC, Rho, Zeff, DEI, attenuation, and spectral slope	Spectral/material-specific quantitative Adipose-containing multivariable model	syngo.via VB40B and UAI.OCR; two senior readers, consensus resolution; no formal reliability coefficient
**Zhang et al., 2025 [37] (Front Neurol)** ^b^	Siemens SOMATOM Drive; third-generation DS-DECT head-and-neck CTA at 80/140 kVp	0.6-mm Br40 reconstructions; VNC, Rho/Z, and 40- and 90-keV monoenergetic images	One intraplaque ROI and two averaged PVAT ROIs; intraplaque and PVAT FF, VNC, IC, Rho, Zeff, attenuation, and spectral slope	Spectral/material-specific quantitative Adipose-containing multivariable model	syngo.via VB40B; two senior readers, consensus resolution; no formal reliability coefficient
**Zhang et al., 2025 [20] (Neuroradiology)** ^b^	Siemens SOMATOM Definition Flash (training) and SOMATOM Drive (external validation); second- and third-generation DS-DECT carotid CTA	Liver VNC, Rho/Z, monoenergetic, and NLB reconstructions; attenuation measured at 40 and 90 keV	One intraplaque ROI and two averaged PVAT ROIs placed at least 1 mm from the carotid lumen and adjacent tissues; intraplaque and PVAT FF, VNC, IC, Rho, Zeff, attenuation at 40 and 90 keV, and spectral slope	Spectral/material-specific quantitative Adipose-containing multivariable model	syngo.via VB40B and UAI.OCR 1.0.8962.1961; two senior radiologists, consensus resolution; no formal reliability coefficient
**Chen et al., 2025 [19] (Jpn J Radiol)**	Philips IQon; DLSCT carotid CTA from the aortic arch to the circle of Willis at 120 kVp	0.9-mm-thick/0.5-mm-increment 40- and 70-keV VMI and polyenergetic images	Semiautomatic plaque-centered carotid PVAT within a radial distance equal to the mean vessel diameter; energy-specific thresholds of −190 to −30 HU on polyenergetic images, −280 to −40 HU at 40 keV, and −220 to −30 HU at 70 keV; PVATPI, PVAT40keV, and PVAT70keV	Conventional quantitative Spectral/material-specific quantitative	IntelliSpace Portal v10.1.1 and ShuKun (ShuKun Technology, Beijing, China); two blinded neuroradiologists with consensus resolution; interobserver ICCs 0.855–0.860 for PVAT attenuation
**Zheng et al., 2026 [23] (Insights Imaging)**	GE Revolution Apex; GSI rapid kVp-switching dual-energy head-and-neck CTA	Conventional polyenergetic images, 40–140-keV VMI, and fat, iodine, and Zeff maps; 0.625-mm thickness/0.5-mm interval	Two 2.5-mm^2^ PVAT ROIs at the level of maximal stenosis, placed at least 1 mm from the vessel boundary and adjacent structures; PVAT defined as −190 to −30 HU on polyenergetic images; ROIs transferred to fat, iodine, Zeff, and 40-/70-keV images for FF, IC, Zeff, attenuation, and spectral-slope measurements	Conventional quantitative Spectral/material-specific quantitative Adipose-containing multivariable model	Advantage Workstation 4.7 and Medis Suite/QAngio CT; two readers blinded to clinical data; interobserver ICCs 0.758–0.865 across plaque and PVAT measurements
**Mundt et al., 2023 [17] (BMC Med Imaging)**	Siemens NAEOTOM Alpha; ECG-gated unenhanced cardiac PCCT at 120 kVp	2-mm Bv40 reconstructions	Manual thoracic descending-aortic PAAT segmentation, −195 to −45 HU; mean attenuation, volume, and 106 radiomic features	Conventional quantitative Radiomic/texture	3D Slicer 4.11, PyRadiomics 3.0.1, and R; expert review, no quantitative repeatability assessment
**Mundt et al., 2024 [41] (Diagnostics)**	Siemens NAEOTOM Alpha; ECG-gated unenhanced cardiac PCCT at 120 kVp	2-mm Bv40 unenhanced axial reconstructions for whole-EAT analysis	Manual whole-EAT segmentation, −190 to −30 HU; radiomics, density, and volume	Conventional quantitative Radiomic/texture	3D Slicer, PyRadiomics, and R; no formal segmentation or radiomics repeatability assessment
**Kahmann et al., 2024 [42] (Insights Imaging)**	Siemens NAEOTOM Alpha PCCT; noncontrast CAC CT plus contrast-enhanced CCTA	0.6-mm-thick/0.4-mm-increment Bv40 CCTA plaque reconstructions; 70-keV Qr40 images for EAT assessment	Manual plaque segmentation for radiomics; EAT density and thickness measured separately	Conventional quantitative Spectral/material-specific quantitative	3D Slicer 4.11, PyRadiomics 3.0.1, Horos PACS 3.3.6, and R Statistics 4.2.0, and RStudio 2022.07.1+554; no formal EAT or radiomics repeatability assessment
**Kahmann et al., 2024 [43] (Front Cardiovasc Med)**	Siemens NAEOTOM Alpha PCCT; noncontrast CAC CT plus contrast-enhanced CCTA	3-mm Qr36 CAC images; 0.6-mm/0.4-mm Bv40 CCTA	Manual LAD/RCA PCAT segmentation, −190 to −30 HU within one vessel diameter; radiomics and mean attenuation	Conventional quantitative Radiomic/texture	3D Slicer 4.11, PyRadiomics 3.0.1, and R; senior review, no quantitative reliability assessment
**Kravchenko et al., 2024 [18] (Eur J Radiol)**	Siemens NAEOTOM Alpha; ECG-gated PCCT CCTA at 120 kVp; paired clinical EID-CT CCTA (SOMATOM Force) at 90–130 kVp	PCCT down-sampled 0.6/0.4-mm Bv40 and UHR 0.2/0.2-mm Bv40/Bv48/Bv64 reconstructions with QIR 4; EID-CT 0.6/0.4-mm Bv40 with ADMIRE 3	Semiautomatic whole-EAT segmentation from the pulmonary trunk to the cardiac apex, −190 to −30 HU; pericardial contours at ∼1-cm intervals with interpolation; one PCD-UHRBv48 mask propagated across PCD reconstructions; EAT volume and mean attenuation	Conventional quantitative	syngo.via VB70 and syngo.via Frontier CT Cardiac Risk Assessment; one board-certified radiologist blinded to medical history; paired cross-system/reconstruction correlation and Bland–Altman analyses; no reader-repeatability coefficient reported
**Zhan et al., 2021 [44] (Jpn J Radiol)**	Siemens SOMATOM Force; third-generation DS-DECT CCTA at 90/Sn150 kVp	Liver VNC reconstructions; CAC and CCTA datasets for cardiac adipose assessment	Three liver ROIs for hepatic fat fraction; threshold-based EAT and total three-vessel PCAT segmentation, −190 to −30 HU	Conventional quantitative Spectral/material-specific quantitative	syngo.via 20, MMWP, and VB20; Bland–Altman analysis showed good intra-/interobserver agreement
**Li et al., 2025 [46] (Clin Radiol)**	GE Revolution CT; conventional 100-kV CCTA plus 80/140-kVp rsDECT abdominal contrast-enhanced CT	0.625-mm thin-slice abdominal images; VUE liver-fat maps generated using MMD	MMD-derived hepatic fat volume fraction; threshold-based EAT and three-vessel PCAT volume and attenuation, −190 to −30 HU	Spectral/material-specific quantitative Adipose-containing multivariable model	AW4.7 MMD and Skview; no study-specific repeatability coefficient
**Dang et al., 2026 [45] (Insights Imaging)**	Philips IQon; DLSCT CCTA and noncontrast abdominal CT	120-kVp-equivalent images, 40- and 70-keV VMI, and Eff-Z maps	Semiautomatic L3 VAT segmentation, −190 to −30 HU, propagated across spectral maps; area, CTpoly, CT40keV, CT70keV, spectral slope, and Eff-Z	Conventional quantitative Spectral/material-specific quantitative Adipose-containing multivariable model	IntelliSpace Portal v6.5; blinded dual reading; all sampled intra-/interobserver ICCs >0.80

*Note*. Only technical elements relevant to biomarker derivation and cross-study comparability are shown. Biomarker derivation categories refer to eligible adipose biomarkers and are non-mutually exclusive where applicable. “Conventional quantitative” refers to conventional measurements derived from eligible advanced CT datasets, whereas “spectral/materialspecific quantitative” refers to measurements specifically derived from spectral or material-specific information. ^a^ Chen et al. (2021) [35], Dang et al. (2021) [21], and Zhu et al. (2021) [36] were considered one likely overlapping coronary DLSCT study cluster. ^b^ The three Zhang et al. (2025) [20,22,37] reports (QIMS, Frontiers in Neurology, and Neuroradiology) were considered one likely overlapping carotid DS-DECT study cluster. Abbreviations: CAC, coronary artery calcium; CCTA, coronary computed tomography angiography; CTA, computed tomography angiography; CTpoly, conventional polychromatic CT attenuation; ADMIRE, Advanced Modeled Iterative Reconstruction; DEI, dual-energy index; DLSCT, dual-layer spectral-detector computed tomography; DS-DECT, dual-source dual-energy computed tomography; EID-CT, energy-integrating detector computed tomography; EAT, epicardial adipose tissue; ECG, electrocardiographic; Eff-Z/Zeff, effective atomic number; FAI, fat attenuation index; FF, fat fraction; GSI, gemstone spectral imaging; HU, Hounsfield unit; ICC, intraclass correlation coefficient; IC, iodine concentration; keV, kiloelectron volt; kVp, peak kilovoltage; L3, third lumbar vertebral level; LAD, left anterior descending artery; LCX, left circumflex artery; MMD, multimaterial decomposition; NLB, nonlinear blending; PAAT, periaortic adipose tissue; PCAT, pericoronary adipose tissue; PCD, photon-counting detector; PCCT, photon-counting computed tomography; PI, polyenergetic imaging; PVAT, perivascular adipose tissue; QIR, quantum iterative reconstruction; RCA, right coronary artery; Rho, electron density; ROI, region of interest; rsDECT, rapid kVp-switching dual-energy computed tomography; UHR, ultra-high resolution; VAT, visceral adipose tissue; VMI, virtual monoenergetic image; VNC, virtual noncontrast; VOI, volume of interest; VUE, virtual unenhanced.

### 3.3. Narrative Synthesis of the Principal Findings

The principal quantitative findings and clinically relevant association or performance estimates are summarized in Table 3.

#### 3.3.1. Coronary DLSCT-Derived PCAT Biomarkers

Across six coronary DLSCT reports, PCAT biomarkers were related to coronary plaque phenotype, CAD status, or stenosis severity. Direct comparison was limited by variation in sampling location, adipose thresholds, endpoints, and statistical models [21,35,36,38,39,40].

Chen et al. [35] reported higher plaque-centered PCAT attenuation around high-risk than non-high-risk plaques, with the largest separation at 40 keV (−119.87 versus −153.76 HU; p<0.001). A post hoc threshold derived and tested in the same sample yielded an AUC of 0.883, 72.9% sensitivity, and 99.5% specificity.

Dang et al. [21] found progressively higher lesion-centered FAI at 40 keV (FAI40keV) across stenosis categories; FAI40keV was independently associated with CAD (OR, 1.058; 95% CI, 1.044–1.073; p<0.001). A post hoc cutoff, evaluated in the same dataset, yielded an AUC of 0.811 (95% CI, 0.769–0.848), 75.2% sensitivity, and 83.0% specificity.

Zhu et al. [36] reported differences in lesion-centered FAI40keV, spectral attenuation slope, and effective atomic number among participants with no stenosis, <50% stenosis, or ≥50% stenosis. FAI40keV and spectral slope yielded AUCs of 0.84 and 0.80, respectively, for ≥50% stenosis; these estimates arose from equal-sized, outcome-defined groups and were not validated.

In the propensity-score-matched study by Zou et al. [38], proximal three-vessel FAI40keV was higher in patients with CAD than in controls (−128.93 versus −138.47 HU; p<0.001). A combined spectral PCAT model achieved an apparent AUC of 0.830 without reported calibration or validation.

Jia et al. [39] reported higher peri-plaque FAI on conventional and virtual monoenergetic images (FAICI and FAIVMI) in severe than in mild or moderate stenosis (p<0.05); peri-plaque spectral slopes, proximal FAI, and proximal spectral slopes did not differ significantly by stenosis severity. Proximal and peri-plaque measures differed by plaque type, with the highest peri-plaque values in low-density noncalcified plaques. No diagnostic model was developed.

Song et al. [40] found the highest peri-plaque FAI40keV in noncalcified plaques, followed by mixed and calcified plaques (p<0.001). A model combining FAI40keV, the 40–70-keV spectral attenuation slope, and effective atomic number discriminated high-risk from non-high-risk plaques with an apparent AUC of 0.881, 87.1% sensitivity, and 75.0% specificity in the development dataset.

No coronary PCAT model or threshold was externally validated, and calibration was absent or incompletely reported [21,35,36,38,40]. Because Chen, Dang, and Zhu reports probably included overlapping participants, their concordant findings were not treated as three independent replications [21,35,36].

#### 3.3.2. Carotid DECT-Derived PVAT Biomarkers

In one carotid report (n=69), arteries ipsilateral to acute cerebral infarction had lower PVAT fat fraction and higher 40-keV attenuation than asymptomatic target arteries (both p<0.001) [22].

A model integrating PVAT, plaque composition, and luminal variables achieved an apparent AUC of 0.934, with 95.0% sensitivity and 77.6% specificity in the development sample [22].

A related report (n=75) combined intraplaque and PVAT spectral parameters; the final nomogram yielded an apparent AUC of 0.962, with 95.8% sensitivity and 82.4% specificity [37].

A third report (n=212) evaluated a multidimensional model integrating PVAT, plaque, and serum lipid variables; the model yielded an AUC of 0.832 in the training cohort and 0.810 in the external-validation cohort [20].

In a prospective DLSCT study (n=73), plaque-centered PVAT40keV was higher around symptomatic than asymptomatic plaques and yielded an AUC of 0.864, with 89.7% sensitivity and 72.7% specificity [19].

In a retrospective rsDECT study of 306 patients, Zheng et al. [23] found higher PVAT effective atomic number and iodine concentration and lower fat fraction in symptomatic than asymptomatic carotid atherosclerosis. CT70keV and fat fraction yielded AUCs of 0.857 and 0.820, respectively; adding CT70keV to stenosis severity and necrotic core volume increased apparent discrimination from an AUC of 0.821 to 0.916 (95% CI, 0.884–0.945), with 85.8% sensitivity and 83.9% specificity. No external validation was performed.

The two single-center Zhang models did not undergo external validation, whereas the dual-center model was externally validated. Because the three Zhang reports probably included overlapping participants, they were considered one likely independent cluster rather than three independent replications [20,22,37].

#### 3.3.3. PCCT-Based Adipose-Tissue Attenuation, Volume, and Radiomics

The five PCCT reports were heterogeneous in design and evaluated different adipose or plaque-adipose targets using attenuation, volumetric, and radiomic/texture measures [17,18,41,42,43].

Mundt et al. [17] reported that the PAAT feature ClusterProminence decreased across increasing Agatston score (p=0.019), whereas mean PAAT attenuation did not differ significantly. An exploratory one-feature model yielded a 10-fold cross-validated $R^{2}$ of 0.368.

In a subsequent whole-EAT analysis, four radiomic features differed between patients with coronary calcification (Agatston score, 1–99 or ≥100) and those without calcification. No adjusted diagnostic model, diagnostic AUC, or independent validation was reported [41].

Kahmann et al. [42] evaluated coronary plaque radiomics alongside conventionally measured EAT density and thickness; EAT density was higher in participants with ≥50% stenosis, high-risk plaque features, or the combined high-risk phenotype, whereas EAT thickness did not differ significantly between groups.

In a matched PCAT radiomics study, GrayLevelNonUniformity was lower in participants with than without CAD around both the LAD and RCA. The analysis remained exploratory, without adjusted effect estimates, diagnostic-accuracy assessment, or validation [43].

Kravchenko et al. [18] prospectively compared paired conventional energy-integrating detector CT (EID-CT) and PCCT CCTA examinations in 50 patients. On the PCD-UHRBv48 reconstruction, EAT volume correlated positively with CACS, CAD-RADS, total coronary calcium volume, and total coronary plaque volume, whereas EAT attenuation showed no significant correlations with these coronary burden measures. Cross-system correlations were excellent for EAT volume but only moderate for attenuation, and absolute EAT volume and attenuation values differed across detector and reconstruction settings. These associations were unadjusted, and no diagnostic or predictive model was developed or validated.

None of the four PCCT radiomics reports externally validated a prespecified feature set or complete analysis pipeline [17,41,42,43]; Kravchenko et al. [18] did not develop a diagnostic or predictive model.

#### 3.3.4. DECT Systemic and Ectopic Adiposity Biomarkers

Three reports related DLSCT-, DS-DECT-, or rsDECT-derived measures of hepatic or abdominal adiposity to coronary high-risk plaque or atherosclerotic burden [44,45,46].

Zhan et al. [44] found that hepatic fat fraction increased across the no-plaque, non-high-risk plaque, and high-risk plaque groups (p<0.001) and remained independently associated with high-risk plaque (adjusted OR, 1.93; 95% CI, 1.53–2.45).

Li et al. [46] similarly reported increasing hepatic fat volume fraction across these plaque groups (p<0.001), with an adjusted OR of 2.55 (95% CI, 1.68–3.88); the apparent AUC was 0.931 for hepatic fat volume fraction and 0.968 for a model combining diabetes, high-sensitivity C-reactive protein, and hepatic fat volume fraction.

Dang et al. [45] reported lower L3-level VAT attenuation at 40 keV in participants with than without metabolic syndrome. Within the metabolic-syndrome group, VAT CT40keV was inversely associated with high-risk plaque (adjusted OR, 0.467; 95% CI, 0.311–0.702) and showed modest discrimination (AUC, 0.728).

None of the ectopic-adiposity associations or adipose-containing models was independently validated [44,45,46].

### 3.4. Risk of Bias and Methodological Appraisal

**Figure 4:**
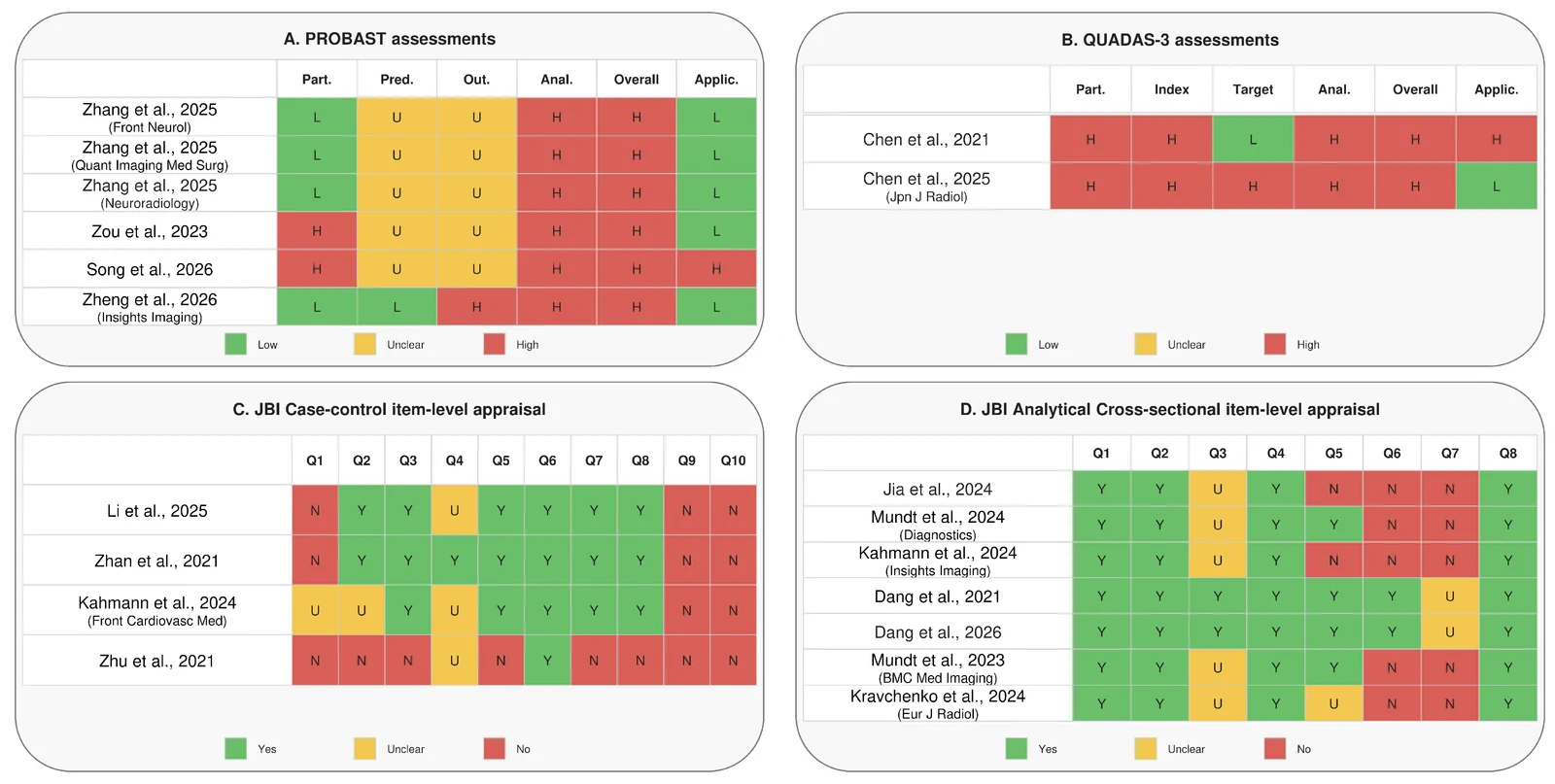
Traffic-light plot of the risk-of-bias and critical-appraisal assessments for the 19 included reports. (**A**) PROBAST domain-level and overall judgments for the six prediction-model reports [20,22,23,37,38,40]; (**B**) QUADAS-3 domain-level, overall risk-of-bias, and applicability judgments for the two eligible diagnostic-accuracy estimates [19,35]; (**C**) item-level appraisal of four case–control reports using the JBI Critical Appraisal Checklist for Case Control Studies [36,43,44,46]; and (**D**) item-level appraisal of seven analytical cross-sectional reports using the revised JBI risk-of-bias tool for analytical cross-sectional studies [17,18,21,39,41,42,45]. For PROBAST and QUADAS-3, green, yellow, and red indicate low, unclear, and high risk of bias or applicability concern, respectively. For the JBI tools, green indicates yes, yellow indicates unclear, and red indicates no. JBI item-level responses are presented without numerical scoring and should not be interpreted as overall low-, moderate-, or high-risk-of-bias classifications. Abbreviations: Anal., Analysis; Applic., Applicability; JBI, Joanna Briggs Institute; Out., Outcome; Part., Participants; Pred., Predictors; PROBAST, Prediction model Risk Of Bias ASsessment Tool; QUADAS-3, Quality Assessment of Diagnostic Accuracy Studies-3.

Methodological appraisal was completed for all 19 reports (Figure 4). Six prediction-model reports were evaluated with PROBAST; the post hoc CT40keV accuracy estimate from Chen et al. [35] and the PVAT40keV accuracy estimate from Chen et al. [19] were evaluated with QUADAS-3, four case–control reports were appraised using the JBI Case Control Checklist, and seven analytical cross-sectional reports were appraised with the revised JBI Analytical Cross-sectional tool. Complete domain- and item-level assessments are reported in Supplementary Tables S3–S6.

All six prediction-model reports were judged to have high overall risk of bias, predominantly because of limitations in the analysis domain; recurrent concerns included few outcome events relative to candidate predictors, data-driven predictor selection, incomplete handling of missing or correlated data, insufficient assessment of calibration and optimism, and absence of external validation in five reports and limited external validation in the dual-center report [20,22,23,37,38,40].

Applicability concern was low for the three Zhang models [20,22,37], the Zou model [38], and the Zheng model [23], but high for the Song model [40] because the plaque spectrum used for high-risk-plaque classification was restricted.

The CT40keV threshold estimate from Chen et al. [35] had high overall risk of bias and high applicability concern because of restrictive participant and plaque selection, post hoc threshold determination in the derivation sample, and failure to account for clustering of multiple plaques within patients. The PVAT40keV estimate from Chen et al. [19] also had high overall risk of bias because of unclear sampling, post hoc cutoff selection, exclusion of uninterpretable scans, and same-sample estimation without validation; applicability concern was low.

Among the eleven reports appraised with JBI tools, inclusion criteria, endpoint definitions, and imaging procedures were generally described adequately. Recurrent limitations were incomplete quantitative repeatability assessment, insufficient identification or control of confounding, contemporaneous exposure and outcome measurement, clustered observations, multiple comparisons, and high-dimensional exploratory feature selection. Dang et al. [21] met the greatest number of items on the revised cross-sectional tool, although the appropriateness of the statistical analysis was unclear. Zhu et al. [36] had broader limitations involving group comparability, matching, differential verification, group-dependent PCAT sampling, confounder control, temporal ordering, and statistical analysis.

All eleven JBI-appraised reports were retained in the qualitative synthesis; retention should not be interpreted as an overall low-risk judgment [17,18,21,36,39,41,42,43,44,45,46].

Table 4 provides a study-level summary of evidence maturity across the four technology–biomarker domains, including the number of reports and likely independent study clusters, repeatability or agreement assessment, adjusted or multivariable analysis, internal and external validation, and the main methodological or risk-of-bias concerns.

### 3.5. Descriptive Synthesis of Reported Performance

Twelve reports presented an AUC for an adipose biomarker, threshold, or adipose-containing multivariable model. Supplementary Figure S1 displays selected study-level estimates descriptively, separating single adipose biomarkers (panel A) from multivariable adipose-containing models (panel B); 95% confidence intervals are shown when reported. Only one displayed estimate represented external validation, whereas the remaining estimates were derived from development or same-sample analyses. No pooled estimate was calculated because adipose depots, CT technologies, target conditions, model composition, populations, and units of analysis differed [19,20,21,22,23,35,36,37,38,40,45,46].

Because the studies differed substantially in adipose depot, CT technology, target condition, population, unit of analysis, and model structure, the displayed AUCs were not interpreted as comparative measures of performance or used to rank biomarkers or models.

## 4. Discussion

The available evidence spanned four domains: coronary PCAT assessed with DLSCT; carotid PVAT assessed with DECT; PCCT-based adipose-tissue attenuation, volume, and radiomic/texture features; and spectral measures of hepatic or visceral adiposity. Three findings define the current evidence. First, advanced CT can extend adipose-tissue assessment beyond volume and mean attenuation to energy-dependent, material-decomposition, and texture measures. Second, these measures appear complementary rather than interchangeable: PCAT and carotid PVAT interrogate the local vessel–adipose interface, whereas EAT, PAAT, hepatic fat, and VAT capture regional or systemic phenotype. Third, the evidence remains exploratory, with predominantly single-center studies and substantial methodological heterogeneity.

The review therefore did not identify a biomarker ready for clinical adoption. Instead, it delineates a translational pathway in which analytical validity and reproducibility must precede biological validation, external model validation, and evaluation of incremental clinical value.

The most coherent signal arose from the six coronary DLSCT reports. Low-keV PCAT attenuation, particularly at 40 keV, and, in some studies, spectral attenuation slope or effective atomic number differed according to high-risk plaque, CAD, or stenosis severity [21,35,36,38,39,40].

These observations are biologically plausible in light of the bidirectional vessel–adipose inflammatory axis and are directionally consistent with conventional FAI literature, including CRISP-CT and a previous meta-analysis [5,7,8].

However, the advanced spectral measurements were not equivalent to the validated conventional FAI construct: studies used different adipose thresholds, energy levels, software, and plaque-centered, lesion-centered, peri-plaque, or proximal-vessel sampling. Apparent enhancement at low keV may partly reflect improved contrast sensitivity, but it may also amplify dependencies on luminal enhancement and reconstruction [9,10,11,12,14]. The reported 40-keV values and cutoffs are therefore protocol-specific and cannot be generalized as standardized markers of vascular inflammation. None was validated against a biological reference standard or prospective cardiovascular outcome [21,35,36,38,39,40].

The five carotid reports suggest that PVAT can be incorporated into multiparametric DECT assessment beyond the coronary circulation [19,20,22,23,37]. Models combining PVAT with plaque, luminal, intraplaque, or serum lipid variables showed high apparent discrimination for symptomatic target arteries or acute ipsilateral cerebral infarction [20,22,37]. One dual-center model retained an AUC of 0.810 in external validation, whereas a prospective DLSCT report found that plaque-centered PVAT40keV discriminated symptomatic from asymptomatic carotid plaques (AUC, 0.864) [19,20]. Zheng et al. [23] extended this evidence to rapid kVp-switching DECT, with an apparent AUC of 0.916 for a model incorporating CT70keV, stenosis severity, and necrotic core volume; however, the result remained derived from a single-center development sample. These findings are consistent with evidence linking pericarotid attenuation to disease stage, cerebrovascular outcomes, and histological vulnerability [25,47,48,49]. Given the probable overlap among three Zhang reports (Supplementary Table S7), these findings support feasibility rather than independent reproducibility, broad transportability, or clinical utility.

The five PCCT reports addressed complementary questions, ranging from radiomic characterization of adipose-tissue heterogeneity to quantitative assessment of EAT volume and attenuation [17,18,41,42,43]. Associations of PAAT and whole-EAT radiomic features with coronary calcium, conventional EAT measures with coronary plaque characteristics, and vessel-specific PCAT radiomic features with CAD support technical feasibility [17,41,42,43]. In the paired study by Kravchenko et al. [18], EAT volume measured on the selected PCD-UHRBv48 reconstruction correlated with CACS, CAD-RADS, total coronary calcium volume, and total coronary plaque volume, whereas EAT attenuation was not significantly associated with these measures; absolute EAT volume and attenuation also varied across detector and reconstruction settings. Radiomic features, however, are outputs of an entire imaging pipeline rather than intrinsic tissue constants. Acquisition, reconstruction, voxel size, segmentation, resampling, discretization, and filtering can all alter feature values. Standardized definitions such as those from the Image Biomarker Standardisation Initiative improve computational consistency but cannot eliminate upstream imaging variability [50].

Phantom and intra-individual studies further show that PCCT radiomic stability depends on acquisition and reconstruction and may differ from energy-integrating CT [51,52]. The reported signatures should therefore be regarded as pipeline-specific hypotheses requiring prespecified, end-to-end external validation.

The hepatic-fat and abdominal-VAT studies addressed systemic metabolic phenotype rather than the local vessel microenvironment. DECT-derived hepatic fat fraction, spectral hepatic fat volume fraction, and low-keV VAT attenuation were associated with high-risk coronary plaque in selected populations with non-alcoholic fatty liver disease or metabolic syndrome [44,45,46].

These findings are consistent with the broader evidence linking ectopic adiposity and EAT burden with coronary disease [4,24], but their biological interpretation differs from that of PCAT or carotid PVAT. Plaque-centered perivascular measurements may reflect local inflammatory remodeling, whereas hepatic and visceral fat more plausibly index insulin resistance, dyslipidemia, and systemic adipose dysfunction. Advanced CT could potentially integrate local and systemic adipose phenotypes in one examination; no included study tested whether such integration improves prediction beyond established clinical and plaque-based measures.

Technical variability is the principal barrier to synthesis and translation. Even studies using the same DLSCT scanner differed in contrast administration, bolus triggering, reconstruction, energy level, adipose thresholds, software, and coronary sampling. Contrast kinetics are especially important because PCAT is vascularized and lies adjacent to an iodine-filled lumen. Dynamic, phantom, and experimental studies demonstrate effects of scan timing, tube voltage, VMI level, luminal enhancement, reconstruction kernel, and iterative reconstruction on PCAT attenuation, apparent volume, and radiomic features [9,10,11,12]. Thus, identical labels such as FAI or PCAT attenuation may denote methodologically different measurements. Absolute HU values, cutoffs, and radiomic features should not be transferred across protocols, reconstruction settings, or scanner platforms without harmonization and validation. The paired EID-CT/PCCT study by Kravchenko et al. [18] further showed that excellent cross-system correlations in EAT volume can coexist with differences in absolute measurements, underscoring the need for protocol- and reconstruction-specific standardization.

Methodological limitations compound this technical heterogeneity. Eighteen reports were single-center, probable cohort overlap reduced the number of independent replications, and every prediction-model report had high overall risk of bias [20,22,23,37,38,40].

Common weaknesses included limited sample or event information, data-driven predictor selection, inadequate control of overfitting, incomplete calibration, and predominant evaluation in development data. Cross-sectional or case–control sampling, residual confounding, multiple comparisons, and clustered observations further limited causal and prognostic inference. Consequently, most reported AUCs represent apparent discrimination in specific samples; the only external-validation estimate came from one dual-center model and does not establish broad transportability, clinical benefit, or comparative superiority.

A plausible future role is therefore adjunctive: spectral PCAT parameters may complement coronary stenosis and plaque assessment; carotid PVAT may complement plaque and luminal characterization; PCCT-derived quantitative and radiomic measures may describe regional adipose tissue and its heterogeneity; and hepatic or visceral measures may provide opportunistic cardiometabolic context. This positioning is consistent with prior reviews of FAI and EAT as complements, rather than alternatives, to anatomical imaging [8,24].

Clinical translation requires a staged validation pathway. Measurement definitions and acquisition and reconstruction protocols should first be prespecified, followed by same-subject repeatability, inter-reader agreement, and cross-scanner and cross-protocol reproducibility. Biological validity should then be tested against histology, molecular imaging, inflammatory markers, or robust plaque phenotypes. Finally, adequately powered multicenter cohorts should assess calibration, external discrimination, incremental value over established benchmarks, and effects on risk classification, treatment decisions, and outcomes. No included biomarker currently supports stand-alone diagnostic or prognostic use.

This review has several strengths. It integrates contemporary DLSCT, DS-DECT, rsDECT, and PCCT evidence across coronary and carotid territories and across local and systemic adipose depots; explicitly evaluates probable cohort overlap; and applies risk-of-bias tools according to study design. Given the substantial differences in imaging protocols, sampling, and endpoints, avoiding quantitative pooling prevented a potentially misleading summary estimate.

The review also has limitations. Searches were restricted to PubMed and Scopus, English-language full-text reports, and publications from January 2020 to September 2026; Embase, Web of Science, grey literature, and trial registries were not included, which may have resulted in relevant studies being missed. Certainty of evidence and reporting bias were not formally assessed because studies did not share a common synthesis. The evidence base was small, technically heterogeneous, geographically concentrated, and predominantly single-center, with only one dual-center report. Relevant evidence may therefore have been missed, and the generalizability of the conclusions is limited.

## 5. Conclusions

Advanced CT can characterize local and systemic adipose phenotype beyond conventional assessment of the vessel lumen and plaque, but the available biomarkers remain investigational. Spectral PCAT parameters and carotid PVAT measures, material-decomposition parameters, and PCCT-derived adipose attenuation, volume, and radiomic features were associated with atherosclerotic phenotypes, while hepatic and visceral fat measures provided complementary cardiometabolic information. These findings derive from heterogeneous, predominantly single-center studies, with external validation limited to one dual-center prediction model, and do not establish clinical utility. Standardized measurement pipelines, biological validation, independent external model validation, and prospective multicenter outcome studies are required to determine whether these biomarkers add value to established clinical and imaging assessment and improve patient management.

## Figures and Tables

**Table 3:** Principal findings and clinical relevance of CT-derived adipose tissue biomarkers.

Study	Population/Technique	Adipose Biomarker	Principal Finding	Clinical Relevance
**Chen et al., 2021 [35] (QIMS)** ^a^	104 patients; 220 plaques; DLSCT CCTA	Plaque-centered PCAT attenuation	CT40keV was higher around HRP than non-HRP (−119.87 vs −153.76 HU; p<0.001)	CT40keV AUC of 0.883 (95% CI, 0.83–0.94); sensitivity of 72.9%; specificity of 99.5%. Post hoc same-sample cutoff; no validation
**Dang et al., 2021 [21] (Front Cardiovasc Med)** ^a^	403 patients; DLSCT CCTA	Lesion-centered PCAT: FAI40keV	FAI40keV increased across stenosis categories and was independently associated with CAD (OR, 1.058; 95% CI, 1.044–1.073; p<0.001)	FAI40keV AUC of 0.811 (95% CI, 0.769–0.848); sensitivity of 75.2%; specificity of 83.0%. Post hoc same-sample cutoff; no validation
**Zhu et al., 2021 [36] (J Cardiothorac Surg)** ^a^	99 selected patients (33 per group); DLSCT CCTA	Lesion-centered PCAT FAI40keV and spectral attenuation slope	FAI40keV, spectral attenuation slope, and Eff-Z differed across no-stenosis, <50%-stenosis, and ≥50%-stenosis groups (p<0.05)	FAI40keV AUC of 0.84 and spectral-slope AUC of 0.80 for ≥50% stenosis. Outcome-based sampling, group-dependent PCAT assessment, and no validation
**Zou et al., 2023 [38] (QIMS)**	90 matched patients; DLSCT CCTA	Proximal three-vessel FAI120kVp and FAIVMI	FAI40keV was higher in CAD than non-CAD patients (−128.93 vs −138.47 HU; p<0.001)	Combined PCAT model AUC of 0.830. Matched development sample; no calibration or independent validation
**Jia et al., 2024 [39] (Front Med)**	160 patients; 231 arteries; DLSCT CCTA	Proximal and peri-plaque FAI on conventional imaging and VMI	Peri-plaque FAI was higher in severe than in mild or moderate stenosis (p<0.05) and was higher in low-density noncalcified and noncalcified plaques than in calcified plaques (p<0.01), with the highest peri-plaque values in low-density noncalcified plaques.	Associations were observed for peri-plaque rather than proximal assessment; no adjusted model, diagnostic AUC, or cutoff was reported
**Song et al., 2026 [40] (BMC Med Imaging)**	182 patients; 312 plaques overall; HRP analysis included 127 patients and 186 plaques; DLSCT CCTA	Peri-plaque PCAT: FAI40keV	FAI40keV decreased from noncalcified to mixed and calcified plaques (−131.3, −146.1, and −153.7 HU; p<0.001)	Combined spectral model AUC of 0.881 (95% CI, 0.833–0.929); sensitivity of 87.1%; specificity of 75.0%. Apparent plaque-level performance; no independent validation
**Zhang et al., 2025 [22] (QIMS)** ^b^	69 patients; third-generation DS-DECT carotid CTA	Carotid PVAT spectral parameters integrated with plaque and lumen variables	Symptomatic arteries showed lower PVAT fat fraction and higher 40-keV attenuation than asymptomatic arteries (both p<0.001)	OACV model AUC of 0.934 (95% CI, 0.872–0.998); sensitivity of 95.0%; specificity of 77.6%. Apparent development performance; no external validation
**Zhang et al., 2025 [37] (Front Neurol)** ^b^	75 patients; third-generation DS-DECT carotid CTA	Carotid PVAT and intraplaque spectral parameters	Combined intraplaque and PVAT features differentiated symptomatic from asymptomatic arteries	Nomo_Model AUC of 0.962 (95% CI, 0.925–1.000); sensitivity of 95.8%; specificity of 82.4%. Apparent development performance; no external validation; likely overlapping Zhang report
**Zhang et al., 2025 [20] (Neuroradiology)** ^b^	212 patients (137 training; 75 external validation); second- and third-generation DS-DECT carotid CTA	Carotid PVAT parameters integrated with plaque characteristics and serum lipid biomarkers	In the training cohort, symptomatic patients showed lower PVAT fat fraction and iodine concentration and higher 40-keV attenuation; these PVAT parameters were retained in the final multidimensional model	External-validation AUC of 0.810 (95% CI, 0.706–0.914); sensitivity of 80.8%; specificity of 65.3%. External validation was performed; however, the model was judged at high risk of bias and likely belongs to the overlapping Zhang study cluster
**Chen et al., 2025 [19] (Jpn J Radiol)**	73 patients; DLSCT carotid CTA	Plaque-centered carotid PVAT attenuation on 40- and 70-keV VMI and polyenergetic images	PVAT40keV was higher around symptomatic than asymptomatic plaques (−70.4±13.4 vs. −96.7±18.7 HU; p<0.001)	PVAT40keV AUC of 0.864 (95% CI, 0.783–0.945); cutoff −86.2 HU; sensitivity of 89.7%; specificity of 72.7%. Post hoc same-sample cutoff; no validation
**Zheng et al., 2026 [23] (Insights Imaging)**	306 patients; rsDECT carotid CTA	Carotid PVAT spectral parameters	Symptomatic patients showed higher PVAT Zeff, IC, CT40keV, and CT70keV and lower FF; Zeff, IC, FF, and CT70keV remained independently associated with symptomatic plaque status	CT70keV AUC of 0.857 (95% CI, 0.815–0.899); FF AUC of 0.820 (95% CI, 0.774–0.865). Stenosis + necrotic core volume + CT70keV model AUC of 0.916 (95% CI, 0.884–0.945); sensitivity of 85.8%; specificity of 83.9%. Apparent development performance; bootstrap resampling was reported, but no external validation
**Mundt et al., 2023 [17] (BMC Med Imaging)**	55 patients; first-generation PCCT	Thoracic PAAT radiomics	ClusterProminence decreased across increasing Agatston score (p=0.019)	Exploratory one-feature model: 10-fold cross-validated $R^{2}$ 0.368; no diagnostic AUC or full-pipeline validation
**Mundt et al., 2024 [41] (Diagnostics)**	53 patients; first-generation PCCT	EAT radiomics	Selected EAT texture features differed across coronary calcium score	Exploratory feature selection; no adjusted model, diagnostic AUC, or independent validation
**Kahmann et al., 2024 [42] (Insights Imaging)**	61 patients; 306 plaques; first-generation PCCT	EAT density and thickness	EAT density was higher with ≥50% stenosis, HRP, and the combined-risk phenotype, whereas EAT thickness was not significant	Exploratory unadjusted subgroup analysis; plaque, rather than EAT, was the radiomic target
**Kahmann et al., 2024 [43] (Front Cardiovasc Med)**	36 matched patients; first-generation PCCT	LAD and RCA PCAT radiomics	GrayLevelNonUniformity was lower in CAD than non-CAD participants in both LAD and RCA PCAT	Exploratory feature selection; no adjusted association, diagnostic AUC, cutoff, or validation
**Kravchenko et al., 2024 [18] (Eur J Radiol)**	50 patients; paired PCCT/EID-CT CCTA	Whole-EAT volume and mean attenuation	On the selected PCD-UHRBv48 reconstruction, EAT volume correlated with CACS (Spearman’s ρ=0.428), CAD-RADS (ρ=0.526), total coronary calcium volume (ρ=0.492), and total coronary plaque volume (ρ=0.373); corresponding EAT attenuation correlations were not significant	Prospective intra-individual comparison; EAT volume showed unadjusted associations with coronary atherosclerotic burden, while absolute EAT volume and attenuation differed across detector and reconstruction settings. No diagnostic or predictive model was developed
**Zhan et al., 2021 [44] (Jpn J Radiol)**	172 patients with NAFLD; third-generation DS-DECT CCTA	Hepatic fat fraction with EAT and PCAT measures	Hepatic fat fraction increased across no-plaque, non-HRP, and HRP groups (6.9%, 8.9%, and 15.3%; p<0.001)	Adjusted OR, 1.93 (95% CI, 1.53–2.45). Case–control association; no primary diagnostic AUC or external validation
**Li et al., 2025 [46] (Clin Radiol)**	159 matched patients with NAFLD; conventional CCTA and rsDECT abdominal CT	Hepatic FVF with EAT and PCAT measures	Hepatic FVF increased across no-plaque, non-HRP, and HRP groups (6.5%, 9.2%, and 13.2%; p<0.001)	FVF OR, 2.55 (95% CI, 1.68–3.88); FVF AUC of 0.931; combined model AUC of 0.968. Apparent performance in a matched retrospective sample; no external validation
**Dang et al., 2026 [45] (Insights Imaging)**	392 patients; DLSCT CCTA and noncontrast abdominal CT	L3 VAT spectral attenuation	VAT CT40keV was lower in patients with metabolic syndrome and, within the metabolic-syndrome group, was independently associated with HRP	Adjusted OR, 0.467 (95% CI, 0.311–0.702); VAT CT40keV AUC of 0.728. Modest discrimination; no independent validation

*Note*. Discrimination estimates were derived in the same analysis or development sample unless otherwise stated; for Zhang et al. [20] (Neuroradiology, 2025), the external-validation estimate is reported. ^a^ Chen et al. (2021) [35], Dang et al. (2021) [21], and Zhu et al. (2021) [36] were considered one likely overlapping coronary DLSCT study cluster. ^b^ The three Zhang et al. (2025) [20,22,37] reports (QIMS, Frontiers in Neurology, and Neuroradiology) were considered one likely overlapping carotid DS-DECT study cluster. Abbreviations: AUC, area under the receiver operating characteristic curve; CAD, coronary artery disease; CAD-RADS, Coronary Artery Disease Reporting and Data System; CACS, coronary artery calcium score; CCTA, coronary computed tomography angiography; CI, confidence interval; CT, computed tomography; DLSCT, dual-layer spectral-detector computed tomography; DS-DECT, dual-source dual-energy computed tomography; EAT, epicardial adipose tissue; EID-CT, energy-integrating detector computed tomography; Eff-Z/Zeff, effective atomic number; FAI, fat attenuation index; FF, fat fraction; FVF, fat volume fraction; HRP, high-risk plaque; HU, Hounsfield unit; IC, iodine concentration; keV, kiloelectron volt; L3, third lumbar vertebral level; LAD, left anterior descending artery; NAFLD, non-alcoholic fatty liver disease; OACV, overall combination variable; OR, odds ratio; PAAT, periaortic adipose tissue; PCAT, pericoronary adipose tissue; PCCT, photon-counting computed tomography; PVAT, perivascular adipose tissue; ρ, Spearman’s rank correlation coefficient; R^2^, coefficient of determination; RCA, right coronary artery; rsDECT, rapid kVp-switching dual-energy computed tomography; VAT, visceral adipose tissue; VMI, virtual monoenergetic image.

**Table 4:** Study-level summary of evidence maturity across the four technology–biomarker domains.

Technology–Biomarker Domain	Study/Report	Repeatability/Agreement	Adjusted/Multivariable Analysis	Internal/External Validation	Main Risk-of-Bias Concerns
**Coronary DLSCT-derived PCAT attenuation or FAI ** *6 reports/4 likely independent study clusters*	**Chen et al., 2021 [35] (QIMS)** ^a^	Interobserver ICC 0.816.	Yes; multivariable logistic analyses evaluated PCAT indicators after adjustment for EAT attenuation and plaque characteristics.	None; post hoc threshold derived and evaluated in the same sample.	High overall RoB (QUADAS-3): highly selected spectrum; post hoc same-sample threshold; plaque clustering not accounted for.
	**Dang et al., 2021 [21] (Front Cardiovasc Med)** ^a^	Triplicate measurements; sampled intraobserver ICCs > 0.75.	Yes; FAI40keV independently associated with CAD.	None; same-sample ROC threshold.	JBI concerns: contemporaneous exposure/outcome; model assumptions and missing-data handling incompletely reported; same-sample ROC threshold.
	**Zhu et al., 2021 [36] (J Cardiothorac Surg)** ^a^	Repeated measurements; intraobserver kappa 0.848.	Yes; multivariable logistic regression identified FAI40keV and spectral attenuation slope as independent predictors of significant stenosis.	None.	JBI concerns: outcome-based sampling; differential verification/group-dependent PCAT sampling; inadequate confounder control.
	**Zou et al., 2023 [38] (QIMS)**	No formal reliability coefficient.	Yes; multivariable combined PCAT model.	None; apparent development performance.	High overall RoB (PROBAST): outcome-dependent matching/restricted sample; incomplete model construction; no calibration or validation.
	**Jia et al., 2024 [39] (Front Med)**	No formal repeatability assessment.	No adjusted model.	Not applicable; no diagnostic/prediction model or AUC.	JBI concerns: PCAT reliability not quantified; confounding and vessel clustering not addressed; multiple comparisons.
	**Song et al., 2026 [40] (BMC Med Imaging)**	Inter-/intraobserver ICCs 0.824–0.939.	Yes; combined spectral PCAT model.	None; apparent plaque-level development performance.	High overall RoB (PROBAST): restricted plaque spectrum; univariable predictor selection; within-patient plaque clustering not addressed.
**Carotid DECT-derived PVAT parameters** *5 reports/3 likely independent study clusters*	**Zhang et al., 2025 [22] (QIMS)** ^b^	Two senior readers; consensus; no formal reliability coefficient.	Yes; multivariable OACV model.	Bootstrap calibration only; no external validation.	High overall RoB (PROBAST): few events; complete-case/data-driven selection; correlated spectral predictors.
	**Zhang et al., 2025 [37] (Front Neurol)** ^b^	Two senior readers; consensus; no formal reliability coefficient.	Yes; multivariable Nomo_Model.	10-fold CV for tuning + bootstrap calibration only; no external validation.	High overall RoB (PROBAST): few events; univariable predictor preselection; incomplete missing-data reporting.
	**Zhang et al., 2025 [20] (Neuroradiology)** ^b^	Two senior radiologists; consensus; no formal reliability coefficient.	Yes; multidimensional multivariable model.	**Bootstrap calibration in development; external validation in a separate-center cohort.**	High overall RoB (PROBAST): data-driven selection/complete-case analysis; predictor blinding unclear; cross-center scanner/protocol heterogeneity.
	**Chen et al., 2025 [19] (Jpn J Radiol)**	Interobserver ICCs 0.855–0.860.	No multivariable model for the selected estimate.	None; post hoc cutoff derived and evaluated in the same sample.	High overall RoB (QUADAS-3): unclear sampling/restricted spectrum; same-sample post hoc cutoff; exclusions/reference-standard concerns.
	**Zheng et al., 2026 [23] (Insights Imaging)**	Interobserver ICCs 0.758–0.865.	Yes; adjusted associations and multivariable CT70keV-containing model.	Bootstrap resampling reported; no optimism-corrected discrimination; no external validation.	High overall RoB (PROBAST): predictor–outcome timing/blinding concerns; missing-data exclusion; data-driven model development.
**PCCT-based adipose attenuation, volume, and radiomic/texture analysis** *5 reports/5 likely independent study clusters*	**Mundt et al., 2023 [17] (BMC Med Imaging)**	Expert review; no quantitative repeatability assessment.	No adjusted model; exploratory one-feature model only.	10-fold CV of one-feature model; no full-pipeline or external validation.	JBI concerns: small sample; 106 candidate features; uncontrolled age/cardiovascular confounding; insufficient feature-selection-pipeline validation.
	**Mundt et al., 2024 [41] (Diagnostics)**	No formal segmentation/radiomics repeatability assessment.	No adjusted diagnostic model.	None; no independent full-pipeline validation.	JBI concerns: small sample; confounding not controlled; data-driven feature selection; no full-pipeline validation.
	**Kahmann et al., 2024 [42] (Insights Imaging)**	No formal EAT/radiomics repeatability assessment.	No; exploratory unadjusted subgroup analysis.	None.	JBI concerns: small overlapping subgroups; no confounder adjustment; extensive multiplicity.
	**Kahmann et al., 2024 [43] (Front Cardiovasc Med)**	Senior review; no quantitative reliability assessment.	No adjusted association/model.	None.	JBI concerns: very small sample; matching incompletely specified; high-dimensional exploratory analysis.
	**Kravchenko et al., 2024 [18] (Eur J Radiol)**	Paired cross-system/reconstruction agreement; no reader-repeatability coefficient.	No; EAT–atherosclerosis associations were unadjusted.	Not applicable; no diagnostic or predictive model developed.	JBI concerns: confounding incompletely addressed; multiple exploratory comparisons/correlations; statistical-analysis concerns.
**Systemic or ectopic adiposity assessed with DECT** *3 reports/3 likely independent study clusters*	**Zhan et al., 2021 [44] (Jpn J Radiol)**	Bland–Altman: good intra-/interobserver agreement.	Yes; adjusted association with high-risk plaque.	None; no external validation.	JBI concerns: residual clinical differences; no temporal ordering.
	**Li et al., 2025 [46] (Clin Radiol)**	No study-specific repeatability coefficient.	Yes; adjusted association and adipose-containing combined model.	None; apparent same-sample performance; no external validation.	JBI concerns: residual group differences; matching insufficiently described; no temporal ordering/matched-analysis concerns.
	**Dang et al., 2026 [45] (Insights Imaging)**	Sampled intra-/interobserver ICCs >0.80.	Yes; adjusted association with high-risk plaque.	None; no independent validation.	JBI concerns: cross-sectional design; incomplete reporting of model assumptions.

*Notes*. Evidence maturity is summarized descriptively and does not constitute a new scoring or grading system. PROBAST and QUADAS-3 overall judgments are reported when available; for JBI-appraised reports, only the principal item-level methodological concerns are summarized, without assigning an overall low-, moderate-, or high-risk category. ^a^ Chen et al. (2021) [35], Dang et al. (2021) [21], and Zhu et al. (2021) [36] were considered one likely overlapping coronary DLSCT study cluster. ^b^ The three Zhang et al. (2025) [20,22,37] reports (QIMS, Frontiers in Neurology, and Neuroradiology) were considered one likely overlapping carotid DS-DECT study cluster. For Kravchenko et al. (2024) [18], paired cross-system/reconstruction agreement was distinguished from reader repeatability. Abbreviations: AUC, area under the receiver operating characteristic curve; CAD, coronary artery disease; CV, cross-validation; DECT, dual-energy computed tomography; DLSCT, dual-layer spectral-detector computed tomography; EAT, epicardial adipose tissue; FAI, fat attenuation index; ICC, intraclass correlation coefficient; JBI, Joanna Briggs Institute; PCAT, pericoronary adipose tissue; PCCT, photon-counting computed tomography; PROBAST, Prediction model Risk Of Bias ASsessment Tool; PVAT, perivascular adipose tissue; QUADAS-3, Quality Assessment of Diagnostic Accuracy Studies-3; RoB, risk of bias.

## Data Availability

The data supporting the findings of this systematic review are provided within the article and its Supplementary Materials. The structured data-extraction workbook and additional methodological appraisal records are available from the corresponding author upon reasonable request.

## Supplementary Materials

The following supporting information can be downloaded at: https://www.mdpi.com/article/10.3390/jcm15207870/s1, Table S1: Complete database search strategies and initial-search log; Table S2: Reports excluded after full-text assessment; Table S3: PROBAST assessments; Table S4: QUADAS-3 assessments; Table S5: JBI Case–control item-level appraisals; Table S6: JBI Analytical cross-sectional item-level appraisals; Table S7: Study-level characteristics supporting the assessment of likely overlapping study cohorts; Table S8: Eligible reported AUC estimates, validation status, and selection for descriptive display; Figure S1: Descriptive display of selected reported AUC estimates; File S1: PRISMA 2020 Main Checklist.

## Abbreviations

The following abbreviations are used in this manuscript:

ADC	Apparent diffusion coefficient
ADMIRE	Advanced Modeled Iterative Reconstruction
ATA	Asymptomatic target artery
AUC	Area under the receiver operating characteristic curve
CAC	Coronary artery calcium
CACS	Coronary artery calcium score
CAD	Coronary artery disease
CAD-RADS	Coronary Artery Disease Reporting and Data System
CCTA	Coronary computed tomography angiography
CI	Confidence interval
CT	Computed tomography
CTA	Computed tomography angiography
CTpoly	Conventional polychromatic CT attenuation
CV	Cross-validation
DECT	Dual-energy computed tomography
DEI	Dual-energy index
DLSCT	Dual-layer spectral-detector computed tomography
DS-DECT	Dual-source dual-energy computed tomography
DWI	Diffusion-weighted imaging
EAT	Epicardial adipose tissue
ECG	Electrocardiographic
Eff-Z/Zeff	Effective atomic number
EID-CT	Energy-integrating detector computed tomography
FAI	Fat attenuation index
FF	Fat fraction
FVF	Fat volume fraction
GSI	Gemstone Spectral Imaging
HFF	Hepatic fat fraction
HRP	High-risk plaque
HU	Hounsfield unit
IC	Iodine concentration
ICC	Intraclass correlation coefficient
JBI	Joanna Briggs Institute
keV	Kiloelectron volt
kVp	Peak kilovoltage
L3	Third lumbar vertebral level
LAD	Left anterior descending artery
LCX	Left circumflex artery
MetS	Metabolic syndrome
MMD	Multimaterial decomposition
MRI	Magnetic resonance imaging
NAFLD	Non-alcoholic fatty liver disease
NLB	Nonlinear blending
OACV	Overall combination variable
OR	Odds ratio
PAAT	Periaortic adipose tissue
PACS	Picture archiving and communication system
PCAT	Pericoronary adipose tissue
PCD	Photon-counting detector
PCCT	Photon-counting computed tomography
PI	Polyenergetic imaging
PRISMA	Preferred Reporting Items for Systematic Reviews and Meta-Analyses
PRISMA-S	PRISMA extension for Searching
PROBAST	Prediction model Risk Of Bias ASsessment Tool
PVAT	Perivascular adipose tissue
QIR	Quantum iterative reconstruction
QUADAS-3	Quality Assessment of Diagnostic Accuracy Studies-3
$R^{2}$	Coefficient of determination
RCA	Right coronary artery
Rho	Electron density
RoB	Risk of bias
ROC	Receiver operating characteristic
ROI	Region of interest
rsDECT	Rapid kVp-switching dual-energy computed tomography
STA	Symptomatic target artery
SWiM	Synthesis Without Meta-analysis
TAVR	Transcatheter aortic valve replacement
TIA	Transient ischemic attack
UHR	Ultra-high resolution
VAT	Visceral adipose tissue
VMI	Virtual monoenergetic imaging
VNC	Virtual noncontrast
VOI	Volume of interest
VUE	Virtual unenhanced
