# Supplementary material for: CT-Derived Adipose Tissue Biomarkers in Coronary and Carotid Atherosclerosis: A Systematic Review of Dual-Energy, Spectral, and Photon-Counting CT
Source: J Clin Med. 2026 Oct 12;15(20):7870. doi: 10.3390/jcm15207870 (PMC13705383; doi:10.3390/jcm15207870)
Supplement: Supplementary file 1 [file jcm-15-07870-s001.zip › jcm-4528626-supplementary.pdf]

SUPPLEMENTARY MATERIALS

CT-Derived Adipose Tissue Biomarkers in Coronary and Carotid Atherosclerosis: A Systematic Review of Dual-Energy, Spectral, and Photon-Counting CT

Supplementary Table S1. Complete database search strategies and search log

Database	Search ID	Search date	Coverage	Records	Exported file
PubMed	PubMed #1 core	June 08, 2026	2020-2026	21	pubmed_01_core_2020_present.nbib
PubMed	PubMed #2 Expanded	June 08, 2026	2020-2026	24	pubmed_02_expanded_2020_present.nbib
PubMed	PubMed #3 Radiomics/plaque	June 08, 2026	2020-2026	69	pubmed_03_radiomics_2020_present.nbib
Scopus	Scopus #1 Core	June 08, 2026	2020-2026	45	scopus_01_core_2020_present.ris
Scopus	Scopus #2 Expanded	June 08, 2026	2020-2026	46	scopus_02_expanded_2020_present.ris
Scopus	Scopus #3 Radiomics/plaque	June 08, 2026	2020-2026	133	scopus_03_radiomics_2020_present.ris

Initial search totals before deduplication: PubMed, 114 records; Scopus, 224 records; overall, 338 records. After removal of 182 duplicates, 156 unique records remained for screening.

S1.1. PubMed #1 core (PubMed)

Search date	Coverage	Records retrieved	Exported file
June 08, 2026	2020-2026	21	pubmed_01_core_2020_present.nbib

("epicardial adipose tissue"[Title/Abstract] OR "pericoronary adipose tissue"[Title/Abstract] OR "perivascular adipose tissue"[Title/Abstract] OR "pericarotid adipose tissue"[Title/Abstract] OR "adipose tissue"[Title/Abstract] OR "visceral adipose tissue"[Title/Abstract] OR EAT[Title/Abstract] OR PCAT[Title/Abstract] OR PVAT[Title/Abstract] OR "fat attenuation index"[Title/Abstract] OR FAI[Title/Abstract])
AND
(atherosclerosis[Title/Abstract] OR "coronary artery disease"[Title/Abstract] OR "coronary atherosclerosis"[Title/Abstract] OR "coronary plaque"[Title/Abstract] OR "carotid atherosclerosis"[Title/Abstract] OR "carotid plaque"[Title/Abstract] OR "carotid stenosis"[Title/Abstract] OR "atherosclerotic plaque"[Title/Abstract])
AND
("spectral CT"[Title/Abstract] OR "spectral computed tomography"[Title/Abstract] OR "dual-energy CT"[Title/Abstract] OR "dual energy CT"[Title/Abstract] OR "dual-energy computed tomography"[Title/Abstract] OR "dual energy computed tomography"[Title/Abstract] OR DECT[Title/Abstract] OR "dual-layer CT"[Title/Abstract] OR "dual layer spectral CT"[Title/Abstract] OR "dual-layer spectral CT"[Title/Abstract] OR "dual-source CT"[Title/Abstract] OR "dual source CT"[Title/Abstract] OR "photon-counting CT"[Title/Abstract] OR "photon counting CT"[Title/Abstract] OR "photon-counting computed tomography"[Title/Abstract] OR "photon counting computed tomography"[Title/Abstract] OR PCCT[Title/Abstract] OR "spectral photon-counting CT"[Title/Abstract] OR "spectral photon counting CT"[Title/Abstract])
AND
("2020/01/01"[Date - Publication] : "3000"[Date - Publication])

S1.2. PubMed #2 Expanded (PubMed)

Search date	Coverage	Records retrieved	Exported file
June 08, 2026	2020-2026	24	pubmed_02_expanded_2020_present.nbib

("epicardial adipose tissue"[Title/Abstract] OR "pericoronary adipose tissue"[Title/Abstract] OR "perivascular adipose tissue"[Title/Abstract] OR "pericarotid adipose tissue"[Title/Abstract] OR "adipose tissue"[Title/Abstract] OR "visceral adipose tissue"[Title/Abstract] OR "fat attenuation index"[Title/Abstract] OR "perivascular fat attenuation"[Title/Abstract] OR "pericoronary fat attenuation"[Title/Abstract] OR "CT fat attenuation"[Title/Abstract] OR "pericoronary inflammation"[Title/Abstract] OR "perivascular inflammation"[Title/Abstract] OR "coronary inflammation"[Title/Abstract] OR EAT[Title/Abstract] OR PCAT[Title/Abstract] OR PVAT[Title/Abstract] OR FAI[Title/Abstract])
AND
(atherosclerosis[Title/Abstract] OR "coronary artery disease"[Title/Abstract] OR "coronary artery atherosclerosis"[Title/Abstract] OR "coronary atherosclerosis"[Title/Abstract] OR "coronary plaque"[Title/Abstract] OR "carotid atherosclerosis"[Title/Abstract] OR "carotid artery disease"[Title/Abstract] OR "carotid plaque"[Title/Abstract] OR "carotid stenosis"[Title/Abstract] OR "plaque vulnerability"[Title/Abstract] OR "vulnerable plaque"[Title/Abstract] OR "high-risk plaque"[Title/Abstract] OR "atherosclerotic plaque"[Title/Abstract])
AND
("spectral CT"[Title/Abstract] OR "spectral computed tomography"[Title/Abstract] OR "dual-energy CT"[Title/Abstract] OR "dual energy CT"[Title/Abstract] OR "dual-energy computed tomography"[Title/Abstract] OR "dual energy computed tomography"[Title/Abstract] OR DECT[Title/Abstract] OR "dual-layer CT"[Title/Abstract] OR "dual layer CT"[Title/Abstract] OR "dual-layer spectral CT"[Title/Abstract] OR "dual layer spectral CT"[Title/Abstract] OR "dual-source CT"[Title/Abstract] OR "dual source CT"[Title/Abstract] OR "photon-counting CT"[Title/Abstract] OR "photon counting CT"[Title/Abstract] OR "photon-counting computed tomography"[Title/Abstract] OR "photon counting computed tomography"[Title/Abstract] OR PCCT[Title/Abstract] OR "spectral photon-counting CT"[Title/Abstract] OR "spectral photon counting CT"[Title/Abstract] OR "multi-energy CT"[Title/Abstract] OR "multi energy CT"[Title/Abstract])
AND
("2020/01/01"[Date - Publication] : "3000"[Date - Publication])

S1.3. PubMed #3 Radiomics/plaque (PubMed)

Search date	Coverage	Records retrieved	Exported file
June 08, 2026	2020-2026	69	pubmed_03_radiomics_2020_present.nbib
(radiomics[Title/Abstract] OR texture[Title/Abstract] OR "radiomic features"[Title/Abstract] OR "plaque characterization"[Title/Abstract] OR "plaque composition"[Title/Abstract] OR "plaque vulnerability"[Title/Abstract] OR "high-risk plaque"[Title/Abstract] OR "vulnerable plaque"[Title/Abstract] OR "virtual monoenergetic"[Title/Abstract] OR "virtual monoenergetic imaging"[Title/Abstract] OR "iodine map"[Title/Abstract] OR "iodine maps"[Title/Abstract] OR "iodine density"[Title/Abstract] OR "effective atomic number"[Title/Abstract] OR Zeff[Title/Abstract])<br>AND<br>(atherosclerosis[Title/Abstract] OR "coronary artery disease"[Title/Abstract] OR "coronary atherosclerosis"[Title/Abstract] OR "coronary plaque"[Title/Abstract] OR "carotid atherosclerosis"[Title/Abstract] OR "carotid plaque"[Title/Abstract] OR "carotid stenosis"[Title/Abstract] OR "atherosclerotic plaque"[Title/Abstract])<br>AND<br>("spectral CT"[Title/Abstract] OR "spectral computed tomography"[Title/Abstract] OR "dual-energy CT"[Title/Abstract] OR "dual energy CT"[Title/Abstract] OR "dual-energy computed tomography"[Title/Abstract] OR "dual energy computed tomography"[Title/Abstract] OR DECT[Title/Abstract] OR "dual-layer CT"[Title/Abstract] OR "dual layer CT"[Title/Abstract] OR "dual-layer spectral CT"[Title/Abstract] OR "dual layer spectral CT"[Title/Abstract] OR "dual-source CT"[Title/Abstract] OR "dual source CT"[Title/Abstract] OR "photon-counting CT"[Title/Abstract] OR "photon counting CT"[Title/Abstract] OR "photon-counting computed tomography"[Title/Abstract] OR "photon counting computed tomography"[Title/Abstract] OR PCCT[Title/Abstract] OR "spectral photon-counting CT"[Title/Abstract] OR "spectral photon counting CT"[Title/Abstract])<br>AND<br>("2020/01/01"[Date - Publication] : "3000"[Date - Publication])			

S1.4. Scopus #1 Core (Scopus)

<table>
<tr><th>Search date</th><th>Coverage</th><th>Records retrieved</th><th>Exported file</th></tr>
<tr><td>June 08, 2026</td><td>2020-2026</td><td>45</td><td>scopus_01_core_2020_present.ris</td></tr>
<tr><td colspan="4">TITLE-ABS-KEY (("epicardial adipose tissue" OR "pericoronary adipose tissue" OR "perivascular adipose tissue" OR "pericarotid adipose tissue" OR "adipose tissue" OR "visceral adipose tissue" OR EAT OR PCAT OR PVAT OR "fat attenuation index" OR FAI)
AND
(atherosclerosis OR "coronary artery disease" OR "coronary atherosclerosis" OR "coronary plaque" OR "carotid atherosclerosis" OR "carotid plaque" OR "carotid stenosis" OR "atherosclerotic plaque")
AND
("spectral CT" OR "spectral computed tomography" OR "dual-energy CT" OR "dual energy CT" OR "dual-energy computed tomography" OR "dual energy computed tomography" OR DECT OR "dual-layer CT" OR "dual layer CT" OR "dual-layer spectral CT" OR "dual layer spectral CT" OR "dual-source CT" OR "dual source CT" OR "photon-counting CT" OR "photon counting CT" OR "photon-counting computed tomography" OR "photon counting computed tomography" OR PCCT OR "spectral photon-counting CT" OR "spectral photon counting CT"))
AND
PUBYEAR > 2019</td></tr>
</table>

S1.5. Scopus #2 Expanded (Scopus)

<table>
<tr><th>Search date</th><th>Coverage</th><th>Records retrieved</th><th>Exported file</th></tr>
<tr><td>June 08, 2026</td><td>2020-2026</td><td>46</td><td>scopus_02_expanded_2020_present.ris</td></tr>
<tr><td colspan="4">TITLE-ABS-KEY (("epicardial adipose tissue" OR "pericoronary adipose tissue" OR "perivascular adipose tissue" OR "pericarotid adipose tissue" OR "adipose tissue" OR "visceral adipose tissue" OR "fat attenuation index" OR "perivascular fat attenuation" OR "pericoronary fat attenuation" OR "CT fat attenuation" OR "pericoronary inflammation" OR "perivascular inflammation" OR "coronary inflammation" OR EAT OR PCAT OR PVAT OR FAI)
AND
(atherosclerosis OR "coronary artery disease" OR "coronary artery atherosclerosis" OR "coronary atherosclerosis" OR "coronary plaque" OR "carotid atherosclerosis" OR "carotid artery disease" OR "carotid plaque" OR "carotid stenosis" OR "plaque vulnerability" OR "vulnerable plaque" OR "high-risk plaque" OR "atherosclerotic plaque")
AND
("spectral CT" OR "spectral computed tomography" OR "dual-energy CT" OR "dual energy CT" OR "dual-energy computed tomography" OR "dual energy computed tomography" OR DECT OR "dual-layer CT" OR "dual layer CT" OR "dual-layer spectral CT" OR "dual layer spectral CT" OR "dual-source CT" OR "dual source CT" OR "photon-counting CT" OR "photon counting CT" OR "photon-counting computed tomography" OR "photon counting computed tomography" OR PCCT OR "spectral photon-counting CT" OR "spectral photon counting CT" OR "multi-energy CT" OR "multi energy CT"))
AND
PUBYEAR > 2019</td></tr>
</table>

S1.6. Scopus #3 Radiomics/plaque (Scopus)

<table>
<tr><th>Search date</th><th>Coverage</th><th>Records retrieved</th><th>Exported file</th></tr>
<tr><td>June 08, 2026</td><td>2020-2026</td><td>133</td><td>scopus_03_radiomics_2020_present.ris</td></tr>
<tr><td colspan="4">TITLE-ABS-KEY ((radiomics OR texture OR "radiomic features" OR "plaque characterization" OR "plaque composition" OR "plaque vulnerability" OR "high-risk plaque" OR "vulnerable plaque" OR "virtual monoenergetic" OR "virtual monoenergetic imaging" OR "iodine map" OR "iodine maps" OR "iodine density" OR "effective atomic number" OR Zeff)
AND
(atherosclerosis OR "coronary artery disease" OR "coronary atherosclerosis" OR "coronary plaque" OR "carotid atherosclerosis" OR "carotid plaque" OR "carotid stenosis" OR "atherosclerotic plaque")
AND
("spectral CT" OR "spectral computed tomography" OR "dual-energy CT" OR "dual energy CT" OR "dual-energy computed tomography" OR "dual energy computed tomography" OR DECT OR "dual-layer CT" OR "dual layer CT" OR "dual-layer spectral CT" OR "dual layer spectral CT" OR "dual-source CT" OR "dual source CT" OR "photon-counting CT" OR "photon counting CT" OR "photon-counting computed tomography" OR "photon counting computed tomography" OR PCCT OR "spectral photon-counting CT" OR "spectral photon counting CT"))
AND
PUBYEAR > 2019</td></tr>
</table>

S1.7. Database update and citation-searching audit trail

Database update (through September 13, 2026). PubMed and Scopus were re-run using the same six search structures shown in S1.1-S1.6 and the unchanged eligibility criteria. The update yielded 47 records before deduplication. Twenty-six duplicate/overlapping records were removed, leaving 21 records; three were already present in the original corpus, leaving 18 new records for title/abstract screening. Seventeen were excluded, one report underwent full-text assessment, and Zheng et al. (2026) was included. The database-search evidence base therefore increased from 17 to 18 included reports.

Citation searching - identification. The 18 reports eligible after the database update served as the seed set. Backward citation searching of their reference lists identified 238 candidate records. Forward citation searching in Scopus for the same 18 reports identified 141 candidate records. Reference lists of the relevant reviews by Sagris et al. (2022), Mancio et al. (2018), and Tuttolomondo et al. (2021) yielded 20 additional candidate records (17, 0 newly advanced, and 3, respectively). The documented citation-searching total was therefore 399 candidate records.

Citation searching - deduplication and prior-record checks. A total of 106 duplicate copies were removed across citation-searching sources: 66 within/backward-search records, 32 within/forward-search records, and 8 cross-method duplicates. A further 32 records were already identified or included in the preceding corpus (30 records from the prior corpus plus Dang et al. 2021 and Zhu et al. 2021). After these steps, 261 new unique citation-searching records remained for screening.

Screening and full-text assessment. The 261 new unique records were screened under the unchanged eligibility criteria, with two reviewers performing title/abstract and full-text decisions and disagreements resolved by consensus. Two hundred fifty-five records were excluded at title/abstract screening and six reports advanced to full-text assessment. Five were excluded - three for wrong CT technology and two for wrong disease/outcome - and one additional report, Kravchenko et al. (2024), was included. The five full-text exclusions are documented in Supplementary Table S2.

Final evidence base. The review therefore included 19 reports. Reports judged to represent probable overlapping cohorts were retained individually for transparency but were not treated as independent replications; the 19 reports represented 15 likely independent study clusters.

Audit note. The finalized project records support the combined-database update totals above, but do not preserve a reliable query-level PubMed/Scopus split for the 47 update records. Query-specific update counts and export filenames were therefore not reconstructed or imputed. The search strings themselves were not redesigned; the six original strategies in S1.1-S1.6 were retained for the update.

Supplementary Table S2. Reports excluded after full-text assessment

Report	Full title	DOI	Primary exclusion reason	Article-specific rationale
Dell'Aversana et al., 2025 (Diagnostics)	Epicardial Adipose Tissue Volume Assessment in the General Population and CAD-RADS 2.0 Score Correlation Using Dual Source Cardiac CT	10.3390/diagnostics15060681	No eligible advanced CT-derived adipose biomarker	Epicardial adipose tissue volume was derived from conventional non-contrast cardiac CT/calcium-scoring images using a standard HU threshold; no DECT, spectral, or PCCT-derived adipose parameter was evaluated.
Shu et al., 2026 (Insights Imaging)	Identification of histological carotid plaque vulnerability by CT angiography using perivascular adipose tissue radiomics signature	10.1186/s13244-025-02134-y	Advanced-CT subgroup not separately extractable	PVAT radiomics was evaluated across conventional CTA and DLSCT acquisitions; results for the DLSCT subgroup were not separately extractable.
Saba et al., 2026 (Eur Radiol)	Photon-counting CT characterization of carotid perivascular adipose tissue: a layer-by-layer quantitative analysis. A preliminary analysis in an asymptomatic population	10.1007/s00330-026-12481-z	No eligible direct atherosclerotic endpoint	PCCT-derived carotid PVAT attenuation was characterized across concentric 1- to 5-mm layers in 20 asymptomatic patients, but no association with plaque phenotype, stenosis severity, symptoms, stroke, or another eligible atherosclerotic endpoint was tested; no longitudinal or clinical outcome data were available.
Jin et al., 2023 (Front Aging Neurosci)	Predicting progression of white matter hyperintensity using coronary artery calcium score based on coronary CT angiography-feasibility and accuracy	10.3389/fnagi.2023.1256228	No eligible advanced CT-derived adipose biomarker; ineligible outcome	Pericoronary FAI was derived from conventional CCTA rather than an eligible spectral/DECT/PCCT adipose dataset. The primary endpoint was white-matter-hyperintensity progression, not an eligible coronary or carotid atherosclerotic endpoint.
Yu et al., 2025 (Radiol Med)	Radiomic phenotype of epicardial adipose tissue derived from coronary artery calcium score predicts myocardial ischemia	10.1007/s11547-025-02063-2	No eligible advanced CT-derived adipose biomarker	EAT radiomics was extracted from conventional coronary artery calcium-scoring images. Although the examination was acquired on a dual-source CT system, no spectral, dual-energy, or photon-counting-derived adipose biomarker was evaluated.
Chen et al., 2025 (Front Cardiovasc Med)	Development of a new threshold for pericoronary fat attenuation based on 40 keV virtual monoenergetic images from dual-energy spectral CT	10.3389/fcvm.2025.1679078	Wrong disease/outcome	The study used spectral detector CT and 40-keV VMI, but its primary aim was methodological: to characterize energy-specific PCAT attenuation and derive a 40-keV threshold. It did not evaluate the adipose biomarker against an eligible coronary or carotid atherosclerotic imaging or clinical endpoint.

Report	Full title	DOI	Primary exclusion reason	Article-specific rationale
Dong et al., 2026 (Comput Methods Programs Biomed)	Predicting OCT-defined vulnerable plaques and MACE using quantitative imaging features of pericoronary adipose tissue from coronary CTA	10.1016/j.cmpb.2026.109570	Wrong CT technology	PCAT radiomic and deep features were extracted from conventional single-energy 120-kVp CCTA on a SOMATOM Definition Flash system. Optical-coherence-tomography-defined plaque vulnerability and major adverse cardiovascular events were relevant outcomes, but the adipose dataset was not generated by an eligible spectral/dual-energy or photon-counting CT acquisition.
Luo et al., 2025 (Eur Radiol)	Additive value of perivascular fat density to CT angiography characteristics of carotid plaques in predicting symptomatic carotid plaques	10.1007/s00330-025-11713-y	Wrong CT technology	Perivascular fat density was measured on conventional single-energy carotid CTA acquired at 100/120 kV. The symptomatic-carotid-plaque endpoint was eligible, but the adipose dataset was not derived from an eligible advanced CT acquisition.
Ma et al., 2023 (Front Endocrinol)	Associations between the serum triglyceride-glucose index and pericoronary adipose tissue attenuation and plaque features using dual-layer spectral detector computed tomography: a cross-sectional study	10.3389/fendo.2023.1166117	Wrong disease/outcome	Although PCAT FAI at 40 keV and spectral attenuation slope were derived from DLSCT, the primary evaluated relationship was with the serum triglyceride-glucose index. Plaque characteristics were analyzed separately, without a direct eligible adipose-biomarker-to-atherosclerosis endpoint analysis.
Yu et al., 2022 (Eur J Radiol)	Associations between pericarotid fat density and image-based risk characteristics of carotid plaque	10.1016/j.ejrad.2022.110364	Wrong CT technology	Pericarotid fat density was derived from conventional single-energy 120-kV carotid CTA. Magnetic-resonance-imaging plaque-risk characteristics were relevant reference findings, but the adipose acquisition did not meet the advanced CT eligibility criterion.

Note. Ten reports were excluded after full-text assessment. Five exclusions arose from the original/database-search workflow: four reports did not meet the advanced-CT adipose-analysis eligibility criterion (including one mixed cohort in which the advanced-CT subgroup was not separately extractable), and one report used eligible PCCT-derived carotid PVAT but did not evaluate a direct atherosclerotic endpoint. Five additional reports were excluded after citation searching: three for wrong CT technology (Dong et al., 2026; Luo et al., 2025; Yu et al., 2022) and two for wrong disease/outcome (Chen et al., 2025; Ma et al., 2023). Kravchenko et al. (2024) was the sixth full-text report assessed after citation searching and was included in the review, so it does not appear in this exclusion table. Jin et al. additionally evaluated an ineligible white-matter-hyperintensity progression endpoint.

Abbreviations: CAD-RADS, Coronary Artery Disease Reporting and Data System; CCTA, coronary CT angiography; CT, computed tomography; CTA, CT angiography; DECT, dual-energy CT; DLSCT, dual-layer spectral-detector CT; DOI, digital object identifier; EAT, epicardial adipose tissue; FAI, fat attenuation index; HU, Hounsfield units; MACE, major adverse cardiovascular events; OCT, optical coherence tomography; PCAT, pericoronary adipose tissue; PCCT, photon-counting CT; PVAT, perivascular adipose tissue; VMI, virtual monoenergetic imaging.

Supplementary Table S3. PROBAST assessments

The final eligible prediction model in each of six reports was assessed using PROBAST.

Study / model	Participants	Predictors	Outcome	Analysis	Overall RoB	Applicability concern
Zhang et al., 2025 (Front Neurol) - Nomo_Model	Low	Unclear	Unclear	High	High	Low
Zhang et al., 2025 (QIMS) - OACV model	Low	Unclear	Unclear	High	High	Low
Zhang et al., 2025 (Neuroradiology) - multidimensional model	Low	Unclear	Unclear	High	High	Low
Zou et al., 2023 (QIMS) - combination model	High	Unclear	Unclear	High	High	Low
Song et al., 2026 (BMC Med Imaging) - combined spectral model	High	Unclear	Unclear	High	High	High
Zheng et al., 2026 (Insights Imaging) - stenosis + necrotic core volume + CT70keV model	Low	Low	High	High	High	Low

Legend: green = Low; yellow = Unclear; orange/red = High.

Supporting rationale

Study	Principal rationale for the final judgments
Zhang et al., 2025 (Front Neurol)	Few outcome events; univariable predictor preselection; incomplete missing-data reporting; no complete optimism correction or external validation.
Zhang et al., 2025 (QIMS)	Only 20 events supported the final model; complete-case analysis; univariable predictor selection; correlated spectral predictors; no external validation or clearly optimism-corrected discrimination.
Zhang et al., 2025 (Neuroradiology)	Dual-center development and external validation, but data-driven predictor selection and complete-case analysis; predictor blinding was unclear; development and validation used different scanners and contrast protocols without formal harmonization; validation was limited to one additional center.
Zou et al., 2023 (QIMS)	Outcome-dependent matching and a restricted sample; model construction incompletely reported; correlated predictors; no calibration or validation; final equation not fully reproducible.
Song et al., 2026 (BMC Med Imaging)	Restricted plaque spectrum; univariable predictor selection; within-patient plaque clustering not addressed; no calibration, optimism correction, or independent validation.
Zheng et al., 2026 (Insights Imaging)	Outcome events could precede CTA by up to 6 months, and outcome-assessor blinding was unreported. Missing data were handled by exclusion; model construction was data-driven, calibration was not reported, and bootstrap resampling did not clearly address the full model-selection process or optimism-corrected performance.

Abbreviations: CT, computed tomography; CTA, CT angiography; LASSO, least absolute shrinkage and selection operator; OACV, overall combination variable; PROBAST, Prediction model Risk Of Bias ASsessment Tool; RoB, risk of bias.

Supplementary Table S4. QUADAS-3 assessments

The assessments apply to two eligible diagnostic-accuracy estimates, one from each report, rather than to every analysis reported in either article.

Estimate	Participants	Index test	Target condition	Analysis	Overall RoB	Applicability concern
Chen et al., 2021 - CT40keV >= -120.60 HU	High	High	Low	High	High	High
Chen et al., 2025 - PVAT40keV ≥ −86.2 HU	High	High	High	High	High	Low

Legend: green = Low; yellow = Unclear; orange/red = High.

Supporting rationale

Study / estimate	Principal rationale for the final judgments
Chen et al., 2021 (QIMS)	Highly selected patient and plaque spectrum; post hoc Youden threshold derived in the same dataset; within-patient clustering of multiple plaques not addressed; sensitivity and specificity estimated in the derivation sample.
Chen et al., 2025 (Jpn J Radiol)	Prospective single-cohort study, but the sampling method was not reported, and eligibility restrictions narrowed the spectrum. The −86.2-HU Youden cutoff was derived and evaluated in the same 73-patient sample; six poor-quality examinations were excluded; the symptomatic-plaque reference definition combined several clinical/imaging pathways without a uniform time window or explicit blinding to the index result; no independent validation was performed.

Abbreviations: CT, computed tomography; HU, Hounsfield units; PVAT, perivascular adipose tissue; QUADAS-3, Quality Assessment of Diagnostic Accuracy Studies 3; RoB, risk of bias.

Supplementary Table S5. JBI Case Control item-level appraisals

Study	Q1	Q2	Q3	Q4	Q5	Q6	Q7	Q8	Q9	Q10	Checklist decision
Li et al., 2025	No	Yes	Yes	Unclear	Yes	Yes	Yes	Yes	No	No	Include
Zhan et al., 2021	No	Yes	Yes	Yes	Yes	Yes	Yes	Yes	No	No	Include
Kahmann et al., 2024 (Front Cardiovasc Med)	Unclear	Unclear	Yes	Unclear	Yes	Yes	Yes	Yes	No	No	Include
Zhu et al., 2021	No	No	No	Unclear	No	Yes	No	No	No	No	Include

Legend: green = Yes; yellow = Unclear; orange/red = No; blue = checklist decision. “Include” denotes retention in the qualitative synthesis and is not an overall low-risk judgment.

Supporting rationale

Study	Main methodological concerns
Li et al., 2025	Residual group differences; matching was insufficiently described; no temporal ordering; matched sampling was not clearly addressed in the analysis.
Zhan et al., 2021	Residual clinical differences and no temporal ordering; matching and adjusted analysis were otherwise adequately reported.
Kahmann et al., 2024 (Front Cardiovasc Med)	Very small sample; matching incompletely specified; radiomic measurement reliability not demonstrated; high-dimensional exploratory analysis without independent validation.
Zhu et al., 2021	Outcome-based equal-group sampling without documented matching; differential verification of stenosis; group-dependent PCAT sampling; inadequate confounder control; no temporal ordering; inconsistent statistical reporting; no validation.

Official item key

Item	Official question
Q1	Were the groups comparable other than the presence of disease in cases or the absence of disease in controls?
Q2	Were cases and controls matched appropriately?
Q3	Were the same criteria used for identification of cases and controls?
Q4	Was exposure measured in a standard, valid and reliable way?
Q5	Was exposure measured in the same way for cases and controls?
Q6	Were confounding factors identified?
Q7	Were strategies to deal with confounding factors stated?
Q8	Were outcomes assessed in a standard, valid and reliable way for cases and controls?
Q9	Was the exposure period of interest long enough to be meaningful?
Q10	Was appropriate statistical analysis used?

Abbreviations: JBI, Joanna Briggs Institute; PCAT, pericoronary adipose tissue.

Supplementary Table S6. JBI Analytical Cross-sectional item-level appraisals

Study	Q1	Q2	Q3	Q4	Q5	Q6	Q7	Q8	Checklist decision
Jia et al., 2024	Yes	Yes	Unclear	Yes	No	No	No	Yes	Include
Mundt et al., 2024	Yes	Yes	Unclear	Yes	Yes	No	No	Yes	Include
Kahmann et al., 2024 (Insights Imaging)	Yes	Yes	Unclear	Yes	No	No	No	Yes	Include
Dang et al., 2021	Yes	Yes	Yes	Yes	Yes	Yes	Unclear	Yes	Include
Dang et al., 2026	Yes	Yes	Yes	Yes	Yes	Yes	Unclear	Yes	Include
Mundt et al., 2023	Yes	Yes	Unclear	Yes	Yes	No	No	Yes	Include
Kravchenko et al., 2024 (Eur J Radiol)	Yes	Yes	Unclear	Yes	Unclear	No	No	Yes	Include

Legend: green = Yes; yellow = Unclear; orange/red = No; blue = checklist decision. "Include" denotes retention in the qualitative synthesis and is not an overall low-risk judgment.

Supporting rationale

Study	Main methodological concerns
Jia et al., 2024	PCAT reliability not quantitatively demonstrated; confounding not addressed; vessel-level clustering and repeated measurements not accounted for; multiple comparisons.
Mundt et al., 2024 (Diagnostics)	Small sample; radiomic repeatability not demonstrated; confounding identified but not controlled; data-driven feature selection and no independent validation of the full pipeline.
Kahmann et al., 2024 (Insights Imaging)	Small, overlapping subgroups; EAT reliability not demonstrated; no confounder adjustment; extensive multiplicity.
Dang et al., 2021	Retrospective single-center design; exposure and outcome measured contemporaneously; lesion-anchored PCAT sampling; model assumptions and missing-data handling incompletely reported; same-sample ROC threshold without validation.
Dang et al., 2026	VAT measurement was reproducible, and the main association was adjusted; residual concerns involved the cross-sectional design, incomplete reporting of model assumptions, and no independent validation.
Mundt et al., 2023 (BMC Med Imaging)	Small sample; 106 candidate features; repeatability not demonstrated; age and cardiovascular confounding not controlled; insufficient validation of the feature-selection pipeline.
Kravchenko et al., 2024 (Eur J Radiol)	Reader repeatability was not quantified; potential confounding of the EAT–atherosclerosis associations was incompletely addressed and no adjusted analysis was performed; multiple exploratory comparisons and correlations raised statistical-analysis concerns.

Official item key

Item	Official question
Q1	Were the criteria for inclusion in the sample clearly defined?
Q2	Were objective, standard criteria used for measurement of the condition?
Q3	Was the exposure measured in a valid and reliable way?
Q4	Were the outcomes measured in a valid and reliable way?
Q5	Were confounding factors identified?
Q6	Were strategies to deal with confounding factors stated?
Q7	Was appropriate statistical analysis used?
Q8	Were the study subjects and setting described in detail?

Abbreviations: EAT, epicardial adipose tissue; JBI, Joanna Briggs Institute; PCAT, pericoronary adipose tissue; ROC, receiver operating characteristic; VAT, visceral adipose tissue.

Supplementary Table S7. Study-level characteristics supporting the assessment of likely overlapping study cohorts

Report	Institution / recruitment setting	Recruitment period	Ethics approval	CT platform	Sample	Main endpoint	Evidence supporting likely overlap
**Likely overlapping coronary DLSCT cluster: Chen 2021 / Dang 2021 / Zhu 2021 (3 reports; treated as 1 likely independent study cluster)**							
Chen et al., 2021 (QIMS)	Shengjing Hospital of China Medical University, Shenyang	Aug 2018–Jan 2020	Shengjing Hospital ethics committee; 2020PS231K	Philips IQon Spectral CT; DLSCT CCTA	104 patients; 220 plaques	CCTA-defined high-risk coronary plaque	Same center, ethics ID and IQon platform; period overlaps Dang/Zhu; shared authors; related spectral PCAT methods.
Dang et al., 2021 (Front Cardiovasc Med)	Shengjing Hospital of China Medical University, Shenyang	Apr 2018–Jul 2020	Shengjing Hospital IRB; 2020PS231K	Philips IQon; DLSCT CCTA	403 patients	Presence of CAD and coronary stenosis severity	Same center, ethics ID and IQon platform; period encompasses Chen/Zhu; extensive author overlap; related spectral PCAT/FAI methods.
Zhu et al., 2021 (J Cardiothorac Surg)	Shengjing Hospital of China Medical University, Shenyang	May 2019–Sep 2019	Shengjing Hospital ethics committee; 2020PS231K	Philips IQon Spectral CT; DLSCT CCTA	99 patients (33/33/33 by stenosis group)	Coronary stenosis category; significant stenosis ≥50%	Same center, ethics ID and IQon platform; interval lies within Chen/Dang periods; shared authors; related spectral PCAT methods.
**Likely overlapping carotid DS-DECT cluster: three Zhang 2025 reports (3 reports; treated as 1 likely independent study cluster)**							
Zhang et al., 2025 (QIMS)	Xuzhou Medical University Affiliated Jiawang District People's Hospital, Xuzhou	Jan 2023–Sep 2024	Affiliated Hospital of Xuzhou Medical University; XYFY2024-KL456	Siemens SOMATOM Drive; third-generation DS-DECT	69 patients (STA 20; ATA 49)	Ipsilateral anterior-circulation acute infarction on MRI DWI/ADC (STA vs ATA)	Substantial overlap in authors and Xuzhou institutional affiliations; recruitment period overlaps both other Zhang reports; SOMATOM Drive also used in the Frontiers report and in the Neuroradiology validation cohort; closely related plaque/PVAT spectral methods; same MRI-defined endpoint.
Zhang et al., 2025 (Front Neurol)	Recruitment hospital not explicitly stated; author affiliations include The Affiliated Hospital of Xuzhou Medical University, Jiawang District People's Hospital, and Xuzhou New Health Hospital, Xuzhou	Jan 2023–Aug 2024	Affiliated Hospital of Xuzhou Medical University; approval number not reported	Siemens SOMATOM Drive; third-generation dual-source CT	75 patients (STA 24; ATA 51)	Ipsilateral anterior-circulation acute infarction on MRI DWI/ADC (STA vs ATA)	Substantial overlap in authors and Xuzhou institutional affiliations; near-identical recruitment period; same third-generation dual-source platform family; closely related intraplaque/PVAT spectral methods; same MRI-defined endpoint.
Zhang et al., 2025 (Neuroradiology)	Two medical centers in Xuzhou; participating centers and center-to-cohort mapping are not explicitly named in Methods. Author affiliations include Affiliated Hospital of Xuzhou Medical University, Jiawang District People's Hospital, and Xuzhou First People's Hospital	Jan 2023–Oct 2024	Participating-hospital IRB; approval No. "[blinded for peer review]"	Training: SOMATOM Definition Flash; validation: SOMATOM Drive	212 patients: training 137; validation 75	Ipsilateral anterior-circulation acute infarction on MRI DWI/ADC (STA vs ATA)	Substantial overlap in authors and Xuzhou institutional affiliations; recruitment period spans the other Zhang reports; validation cohort uses SOMATOM Drive; closely related plaque/PVAT spectral methods; same MRI-defined endpoint. Dual-center design and differing group counts preclude participant-level confirmation.

Note. Overlap judgments were based on report-level similarities in authorship, institution/recruitment setting, recruitment period, ethics approval, CT platform, sample size, endpoint definition, and biomarker methodology. Corresponding authors were not contacted for participant-level clarification. Overlap was therefore considered likely or probable rather than confirmed. All reports were retained individually for transparency, but reports within each suspected cluster were not treated as independent replications; for descriptive counting, each cluster was conservatively considered one likely independent study cluster.

Abbreviations: ADC, apparent diffusion coefficient; ATA, asymptomatic group; CAD, coronary artery disease; CCTA, coronary computed tomography angiography; DLSCT, dual-layer spectral-detector CT; DS-DECT, dual-source dual-energy CT; DWI, diffusion-weighted imaging; FAI, fat attenuation index; MRI, magnetic resonance imaging; PCAT, pericoronary adipose tissue; PVAT, perivascular adipose tissue; STA, symptomatic group. CT, computed tomography; IRB, institutional review board.

Supplementary Table S8. Eligible reported AUC estimates, validation status, and selection for descriptive display

Twelve of the 19 included reports presented eligible AUC estimates for an adipose-tissue biomarker or an adipose-containing model. All eligible reported estimates are summarized below.

Study / endpoint	Eligible reported AUCs (95% CI)	Validation status	Selection for Fig. S1 / rationale
Chen et al., 2021 (QIMS)<br>HRP vs non-HRP	CTpoly: 0.687 (0.60–0.77)<br>CT40keV: 0.883 (0.83–0.94)<br>Zeff: 0.806 (0.74–0.88)<br>λHU: 0.807 (0.74–0.87)	Same-sample diagnostic performance; Youden-derived threshold; no independent validation.	A: CT40keV — principal low-keV spectral PCAT diagnostic estimate.
Dang et al., 2021 (Front Cardiovasc Med)<br>CAD presence	FAI40keV: 0.811 (0.769–0.848)<br>λHU: 0.756 (0.712–0.798)<br>Eff-Z: 0.646 (0.597–0.693)<br>FAI120kVp: 0.731 (0.685–0.774)<br>EAT attenuation: 0.614 (0.564–0.662)	Same-sample diagnostic performance; optimal cut-offs estimated in the same sample; no independent validation.	A: FAI40keV — principal spectral PCAT estimate.
Zhu et al., 2021 (J Cardiothorac Surg)<br>Significant stenosis (≥50%)	FAI40keV: 0.84 (0.76–0.92)<br>λHU: 0.80 (0.70–0.90)	Same-sample diagnostic performance and cut-off estimation; no independent validation.	A: FAI40keV — principal PCAT estimate for significant stenosis.
Zou et al., 2023 (QIMS)<br>CAD presence	FAIVMI model (FAI40, 50, 60, 70, and 80 keV): 0.8123 (CI NR)<br>FAI120kVp model: 0.7444 (CI NR)<br>λ model (λ40–70, λ40–100, and λ70–100keV): 0.7230 (CI NR)<br>Combined model (FAIVMI + FAI120kVp + λ): 0.8296 (CI NR)	Apparent model performance in a matched development sample; no internal or external validation reported.	B: Combined model — authors' final combined PCAT model.
Song et al., 2026 (BMC Med Imaging)<br>High-risk coronary plaque	FAI120kVp: 0.634 (0.551–0.717)<br>FAI40keV: 0.802 (0.739–0.864)<br>λ40–70keV: 0.796 (0.732–0.861)<br>Eff-Z: 0.792 (0.724–0.860)<br>Combined model (FAI40keV + λ40–70keV + Eff-Z): 0.881 (0.833–0.929)	Same-sample biomarker ROC estimates; combined model apparent development performance; no internal or external validation.	A: FAI40keV; B: combined model — principal single estimate and final model.
Zhang et al., 2025 (QIMS)<br>Symptomatic target artery / acute ipsilateral infarction	FF: 0.774 (0.660–0.889)<br>VNC: 0.804 (0.691–0.917)<br>Rho: 0.761 (0.625–0.897)<br>40KH: 0.865 (0.773–0.956)<br>K: 0.860 (0.766–0.954)<br>OACV (FF + VNC + Rho + 40KH + K + LRV/NCV): 0.934 (0.872–0.998)	Same-sample biomarker ROC estimates; OACV apparent development performance; 1,000-bootstrap calibration only; no external validation.	B: OACV — final multidimensional model; predictor-level AUCs retained here but not plotted.

Study / endpoint	Eligible reported AUCs (95% CI)	Validation status	Selection for Fig. S1 / rationale
Zhang et al., 2025 (Front Neurol)<br>Symptomatic target artery / acute stroke	PA_FF: 0.777 (0.670–0.883)<br>PA_VNC: 0.811 (0.711–0.912)<br>PA_Rho: 0.725 (0.594–0.855)<br>PA_K: 0.839 (0.745–0.932)<br>PA_Model (PA_FF + PA_VNC + PA_Rho + PA_K): 0.913 (0.845–0.980)<br>Nomo_Model (IP_Zeff + IP_40KH + IP_K + PA_FF + PA_VNC + PA_Rho + PA_K): 0.962 (0.925–1.000)	Same-sample biomarker/model performance; 10-fold CV used for LASSO tuning and 1,000-bootstrap calibration; no optimism-corrected AUC or external validation.	B: Nomo_Model — final combined model; predictor-level AUCs retained here but not plotted.
Zhang et al., 2025 (Neuroradiology)<br>Acute ipsilateral cerebral infarction	Final model (HDL + LRV + PA_FF + PA_IC + PA_40KH + IP_Zeff + IP_40KH), training: 0.832 (0.761–0.903)<br>Same final model, external validation: 0.810 (0.706–0.914)	Development performance plus external validation in a separate-center cohort; 1,000-bootstrap calibration also reported in the development cohort.	B: 0.810 external-validation AUC — external estimate preferred over training performance.
Chen et al., 2025 (Jpn J Radiol)<br>Symptomatic carotid plaque	PVAT40keV: 0.864 (0.783–0.945)<br>PVAT70keV: 0.750 (0.632–0.869)<br>PVATPI: 0.714 (0.593–0.836)	Same-sample diagnostic performance; Youden-derived cutoff; no independent validation.	A: PVAT40keV — principal diagnostic estimate.
Zheng et al., 2026 (Insights Imaging)<br>Symptomatic carotid atherosclerosis	FF: 0.820 (0.774–0.865)<br>IC: 0.699 (0.640–0.757)<br>Zeff: 0.805 (0.749–0.861)<br>CT70keV: 0.857 (0.815–0.899)<br>Model C1 (stenosis + necrotic core volume + FF): 0.897 (0.859–0.929)<br>Model C2 (stenosis + necrotic core volume + CT70keV): 0.916 (0.884–0.945)	Same-sample biomarker ROC estimates; model AUCs are apparent. Bootstrap resampling assessed multivariable coefficient/association stability, not optimism-corrected AUC; no external validation.	A: CT70keV; B: Model C2 — CT70keV-based single biomarker and corresponding incremental model.

Study / endpoint	Eligible reported AUCs (95% CI)	Validation status	Selection for Fig. S1 / rationale
Li et al., 2025 (Clin Radiol)<br>Non-HRP and HRP vs no plaque	FVF, non-HRP vs no plaque: 0.778 (0.693–0.863)<br>FVF, HRP vs no plaque: 0.931 (0.880–0.981)<br>Joint model 1 (DM + hs-CRP + FVF), HRP vs no plaque: 0.968 (0.935–1.000)	Same-sample diagnostic/model discrimination in a matched retrospective sample; no internal or external validation.	A: FVF (HRP); B: Joint model 1 — HRP-focused estimates matching the principal plaque-vulnerability endpoint.
Dang et al., 2026 (Insights Imaging)<br>High-risk plaque within MetS	CT40keV (Model C): 0.728 (0.681–0.772)<br>Model D (FRS + CT40keV): 0.728 (0.681–0.771)<br>Model E (CACS + CT40keV): 0.733 (0.687–0.777)<br>Model F (FRS + CACS + CT40keV): 0.732 (0.685–0.775)	Same-sample biomarker/model discrimination; no internal or external validation.	A: CT40keV; B: Model F — principal VAT biomarker and fully combined model.

Note. Only AUC estimates directly attributable to eligible adipose-tissue biomarkers or to models containing at least one eligible adipose parameter are listed. Comparator-only estimates without an adipose parameter were omitted (including Zheng Models A/B, Dang 2026 FRS/CACS-only models, Li DM/hs-CRP-only estimates, and Zhang Front Neurol intraplaque-only estimates). CI NR indicates that a confidence interval was not reported in the original article. Panel A of Supplementary Figure S1 displays selected single-biomarker estimates; Panel B displays selected adipose-containing multivariable models. Selection followed the review Methods: estimates most closely aligned with the report's primary objective and review question were used, the authors' final adipose-containing model was preferred for multivariable analyses, and external-validation estimates were preferred when available. Predictor-level AUCs from reports primarily focused on model development were retained in this table but were not necessarily plotted. Bootstrap or cross-validation used only for tuning, calibration, or coefficient/association stability was not treated as an optimism-corrected AUC unless such performance was explicitly reported. The AUCs are descriptive and should not be interpreted as comparative performance across studies.

Abbreviations: AUC, area under the receiver operating characteristic curve; CACS, coronary artery calcium score; CAD, coronary artery disease; CI, confidence interval; CTpoly, conventional polychromatic CT attenuation; CV, cross-validation; DM, diabetes mellitus; EAT, epicardial adipose tissue; Eff-Z/Zeff, effective atomic number; FAI, fat attenuation index; FF, fat fraction; FRS, Framingham risk score; FVF, fat volume fraction; HDL, high-density lipoprotein; HRP, high-risk plaque; hs-CRP, high-sensitivity C-reactive protein; IC, iodine concentration; IP, intraplaque; K/λHU, spectral attenuation slope; LRV, lipid-rich volume; LRV/NCV, lipid-rich volume to non-calcified plaque volume ratio; MetS, metabolic syndrome; NR, not reported; OACV, overall combination variable; PA, perivascular adipose; PCAT, pericoronary adipose tissue; PI, polyenergetic imaging; PVAT, perivascular adipose tissue; Rho, electron density; VNC, virtual non-contrast. CT, computed tomography; LASSO, least absolute shrinkage and selection operator; ROC, receiver operating characteristic; VAT, visceral adipose tissue; VMI, virtual monoenergetic imaging; 40KH, CT attenuation at 40 keV.

Supplementary Figure S1. Descriptive display of selected reported AUC estimates.

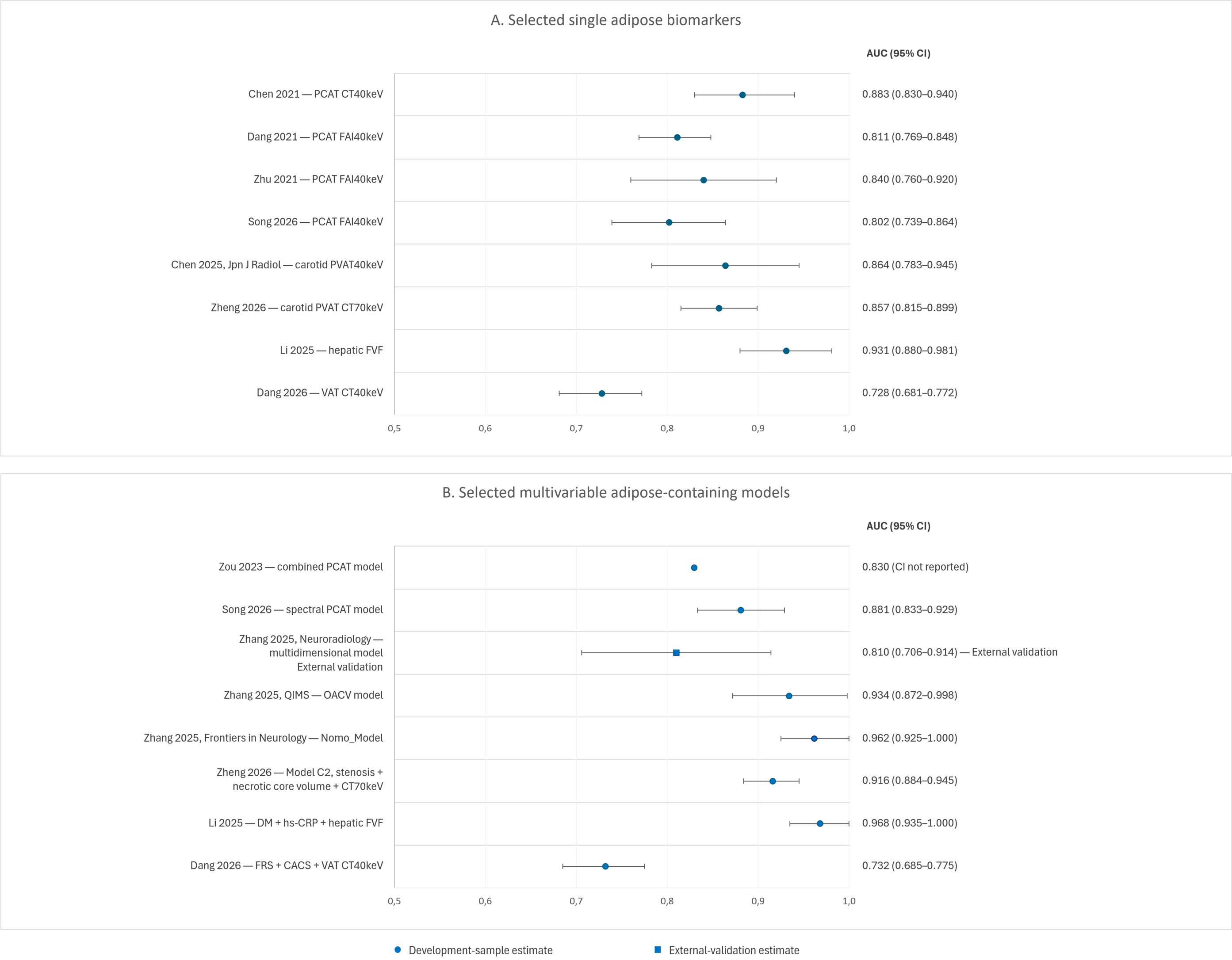

Forest-like plots of reported discrimination performance of CT-derived adipose-tissue biomarkers. (A) Selected individual adipose-tissue biomarkers. (B) Selected multivariable models including at least one eligible adipose-tissue parameter. Points represent reported areas under the receiver operating characteristic curve (AUC), and horizontal bars indicate reported 95% confidence intervals (CI). No 95% CI was reported for the combined model of Zou et al. (2023). For Zhang et al. (Neuroradiology, 2025), the external-validation estimate is shown and explicitly identified. Estimates are presented descriptively and should not be interpreted as comparative performance across biomarkers or models because the studies differed in populations, endpoints, adipose depots, CT technologies, units of analysis, model structure, and validation status.
